# Assessing vaccine strategies for mpox outbreak in New York City using an age-structure model

**DOI:** 10.1186/s12879-024-09551-2

**Published:** 2024-09-30

**Authors:** Zixiao Xiong, Ling Xue, Xuezhi Li, Yanfen Zhang

**Affiliations:** 1 College of Mathematical Sciences, Harbin Engineering University, Harbin, China; 2 School of Mathematics and Information Science, Henan Normal University, Xinxiang, China

**Keywords:** Mpox, Age-structured model, Basic reproduction number, Sensitivity analysis, Vaccination strategies

## Abstract

**Background:**

Since May 7 2022, mpox has been endemic in many countries which has attracted the attention of health authorities in various countries and made control decisions, in which vaccination is the mainstream strategy. However, the shortage of vaccine doses and the reduction of protective efficacy have led to unresolved issues such as vaccine allocation decisions and evaluation of transmission scale.

**Methods:**

We developed an epidemiological model to describe the prevalence of the mpox virus in New York City and calibrated the model to match surveillance data from May 19 to November 3, 2022. Finally, we adjusted the model to simulate and compare several scenarios of non-vaccination and pre-pandemic vaccination.

**Results:**

Relative to the status quo, if vaccination is not carried out, the number of new infections increases to about 385%, and the transmission time will be extended to about 350%, while if vaccinated before the epidemic, the number of new infections decreases to 94.2-96%.

**Conclusions:**

The mpox outbreak in New York City may be linked to the Pride event. However, with current vaccine coverage, there will be no more large-scale outbreaks of mpox, even if there is another similar activity. For areas with limited vaccines, priority is given to high-risk groups in the age group [34–45] years as soon as possible.

**Supplementary Information:**

The online version contains supplementary material available at 10.1186/s12879-024-09551-2.

## Introduction

Mpox virus is an orthopox virus similar to that transmits smallpox and can survive in humans, some animals and fomites [1]. After being infected, 97% of people will develop a rash and more than 50% will develop symptoms such as swollen lymph nodes, headache and fever, in addition to possible rectal bleeding, conjunctivitis, encephalitis and even death [2]. Mpox is a self-limiting disease and the majority of patients usually can recover by the immune response of the body to the mpox virus [3]. However, severe pain from mucosal lesions and scarring lesions may occur [4, 5]. In addition, there is no specific treatment for mpox [6].

Mpox virus was first isolated in Denmark in 1958 from a crab-eating macaque imported from Singapore [7]. Before the discovery of the mpox virus from a 9-year-old child in 1970, it was only found in animals. In the same year, several cases of mpox were diagnosed in Liberia and Sierra Leone caused by infected animals. Besides, there were several outbreaks of mpox in the African region between 1970 and 1997 [8]. Notably, the epidemiology of mpox changed during this period: the mortality rate decreased from 17% to less than 5%, and the proportion of cases infected by animals decreased from 91 to 22% [9]. Mpox virus was prevalent in Africa until 2003 when mpox cases were found in the United States due to animal imports [10]. Afterwards, mpox cases were reported in Israel in 2018, the UK and Singapore in 2020 all from tourists [11–13]. However, in May 2022, no travel history to endemic areas was reported for outbreaks of mpox in Europe and America, which implies that new areas of endemic transmission may have emerged [14]. As of November 17, 2022, the cumulative number of mpox infections has reached 73,087, affecting 108 countries, of which the United States ranks first in the world with 26,778 cases [15]. In June 2023, mpox cases were detected in Beijing and Guangzhou, China. In addition, new mpox cases continue to be reported worldwide [16].

It is worth noting that both global and U.S. cases showed a clear age distribution [2]. For instance, out of the 41,328 cases recorded by the WHO, patients aged 18–29, 30–39, 40–49, 50–59, and 60–69 years are 10,378 (25.1%), 16,618 (40%), 9097 (22%), 3013 (7.2%), and 686 (1.6%), respectively [16]. The main transmission route of this mpox outbreak is human-to-human with the majority being male and having sex with men [16]. The proportion of people infected with the mpox virus without high-risk sexual behavior is about 15% from May 19 to November 3. Therefore, we need to divide the population into high-risk and low-risk categories. Currently vaccines or specific drugs for mpox are not available. Fortunately, the smallpox vaccine provides 85% percent protection against mpox [6, 17]. Since smallpox was eradicated worldwide around 1980, individuals under 40 years old are unprotected [18]. Although people who have previously been vaccinated against smallpox are immune to the mpox virus, the protection rate has fallen below 85% [19, 20]. Obviously, ignoring the above facts will underestimate the scale of mpox transmission. The number of vaccines, including smallpox vaccines and other vaccines, that can be used to prevent mpox virus is limited in some areas [21]. Therefore, exploring how to distribute vaccines efficiently has become a great concern.

## Methods

### Study design

To simulate mpox virus epidemics in New York City, we propose a deterministic epidemiological model to match surveillance data from the first wave of mpox cases from 19 May to 3 November, 2022. We obtain a set of model parameters that best reproduce observed mpox epidemic trends. We then modify our model to assess the impact of not being vaccinated or vaccinated before the pandemic.

### Model structure

We construct a compartmental mpox virus transmission model that takes into account the division of risk groups, the distribution of age, the dynamics of vaccination, and the weakening of vaccine efficacy. Notably, currently 98% of monkeypox patients are male, and more than 95% of them are men who have sex with men [15]. Therefore, we classify all populations as high- and low-risk, with bisexuals and heterosexuality males being high-risk populations. Additionally, the WHO data show a clear age distribution of mpox cases in high-risk populations [15]. We further divide the high-risk population into $$n$$ age-groups as $$[{x}_{1},{x}_{2}),\cdots ,[{x}_{i},{x}_{i+1}),\cdots$$$$[{x}_{n},{x}_{n+1})$$, where$${x}_{i}\in \left[0\hspace{0.25em}{\infty }\right]$$ and $$i,n\in \mathbb{N}$$. Then, in the ith age group $$[{x}_{i},{x}_{i+1})$$, the high-risk population is classified into seven compartments: susceptible ($${S}_{i}^{h}$$), previously vaccinated $$({\stackrel{\sim}{V}}_{i}^{h})$$, currently vaccinated $$\left(\overline{V}{}_{i}^{h}\right)$$ exposed $$\left({E}_{i}^{h}\right)$$, prodromal ($${P}_{i}^{h}$$), infected $$\left({I}_{i}^{h}\right)$$, and recovered ($${R}_{i}^{h}$$). The model describes the dynamics of each compartment. The Details are provided in Sect. 1 of the Supplementary Appendix.

### Surveillance data

The surveillance data we used as a reference for the calibration model are taken from the reports of New York City Health (NYCH). Surveillance data provide weekly new cases of mpox infection from 19 May to 3 November, 2022. These reports also provide an age distribution of the total number of new cases, based on which we assign these cases accordingly in the model. In addition, surveillance data includes daily vaccination doses from 19 May to 3 November 2022. Details are provided in Sect. 2 of the Supplementary Appendix.

### Model calibration

**Fig. 1:**
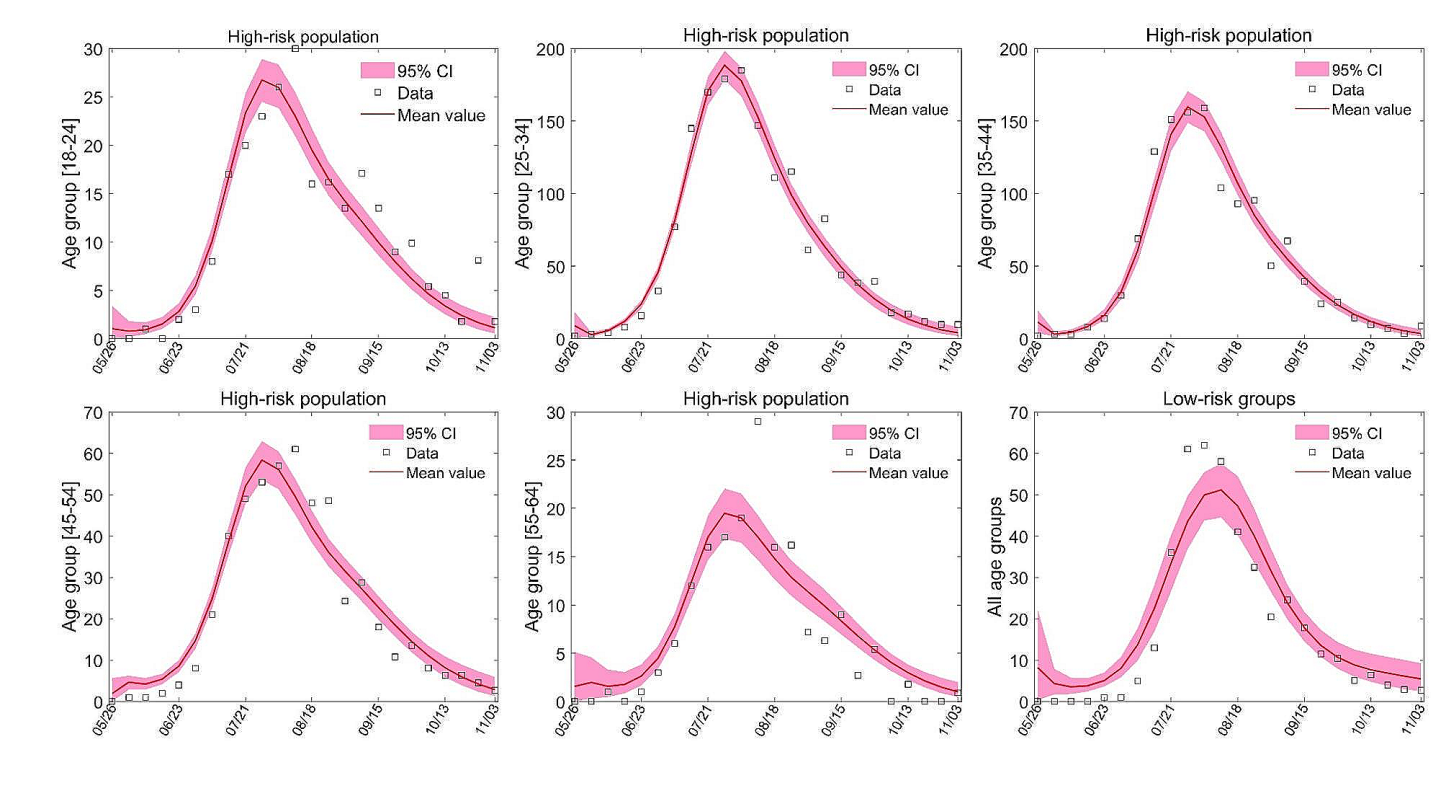
Fitted results of the number of new weekly mpox cases from May 26 to November 23. The solid red line shows the fitted curve of the mpox virus transmission model. The 95% confidence interval is indicated in light red. The white squares represent the actual number of new weekly cases

In this section, we briefly summarize how to calibrate the model. We first provide initial conditions and the range of uncertainty parameters based on literature reviews. To calibrate our model, we use the Markov chain Monte Carlo (MCMC) method to match the model output with NYCH monitoring data from May 19 to November 3, 2022. We randomly select 10% as the final distribution of the parameters after running 800,000 simulations based on the parameter ranges. Figure 1 shows the final fitting result. The Details are provided in Sect. 3 of the Supplementary Appendix.

### Effective reproduction number

We derive the effective reproduction number ${R}_{e}\left(t\right)$ of the model, which can reflect the average number of second-generation cases transmitted by an infected individual during their infection period at a certain moment $t$. We can use ${R}_{e}\left(t\right)$ to measure the real-time transmissibility of the epidemic, and when ${R}_{e}\left(t\right)>1$, the epidemic will spread rapidly in the population. As the ${R}_{e}\left(t\right)$ value gradually decreases, the development speed of the epidemic will gradually decrease, and finally when the ${R}_{e}\left(t\right)<1$, the epidemic gradually disappears. The numerical result of the effective reproduction number of mpox is shown in Figure S10. We find that the effective reproduction number is greater than 1 from June 10th to July 14th and is the largest on June 28th. The New York City Pride on June 25, 2022, may have increased human contacts, and contributed to the epidemic of the mpox virus. The Details are provided in Sect. 4 of the Supplementary Appendix.

### Sensitivity analysis

We perform sensitivity analyses of vaccination rates to determine the effect of vaccination in different age groups on model outcomes. The effective reproduction number is an important indicator to describe the speed and degree of the spread of infectious diseases, and reducing the effective reproduction number is the key to controlling the spread of the epidemic. Therefore, investigating the sensitivity of the effective reproduction number with respect to vaccination rates can obtain parameters for effective control of the spread of the epidemic. Moreover, in order to more visually show the impact of vaccination rate on the number of new confirmed cases, we also investigate the sensitivity of the cumulative number of new cases to the vaccination rate. The results of the sensitivity analysis are shown in Figure S11 which shows that the vaccine is negatively correlated with the effective reproduction number and cumulative number of new cases. Meanwhile, the effective number of reproductions and the cumulative number of new cases are sensitive to vaccination rates in high-risk individuals aged 35–44 years, which means that vaccination in this age group is most effective in reducing the spread of the epidemic. In particular, the correlation between the effective vaccination rate and the effective reproduction number gradually decreases with time. It is evident that vaccination administered earlier produces a more noticeable effect. Vaccine hesitancy will exacerbate the public health and economic burden. This means that if people do not get vaccinated during the early stages of an epidemic, more vaccines will be required later to achieve the same outcome. The Details are provided in Sect. 5 of the Supplementary Appendix.

## Results

Vaccination is regarded as the most effective measure to control infectious diseases. The NYCH released on the number of daily vaccinations starting from May 22. As of November 3, 100,470 individuals have received the first dose of the vaccine in New York City. We assume that after the first vaccination, all the individuals will produce antibodies, i.e., 85% protection against mpox. In the following, the impact of non-vaccination and early vaccination on mpox outbreaks was assessed.

**Fig. 2:**
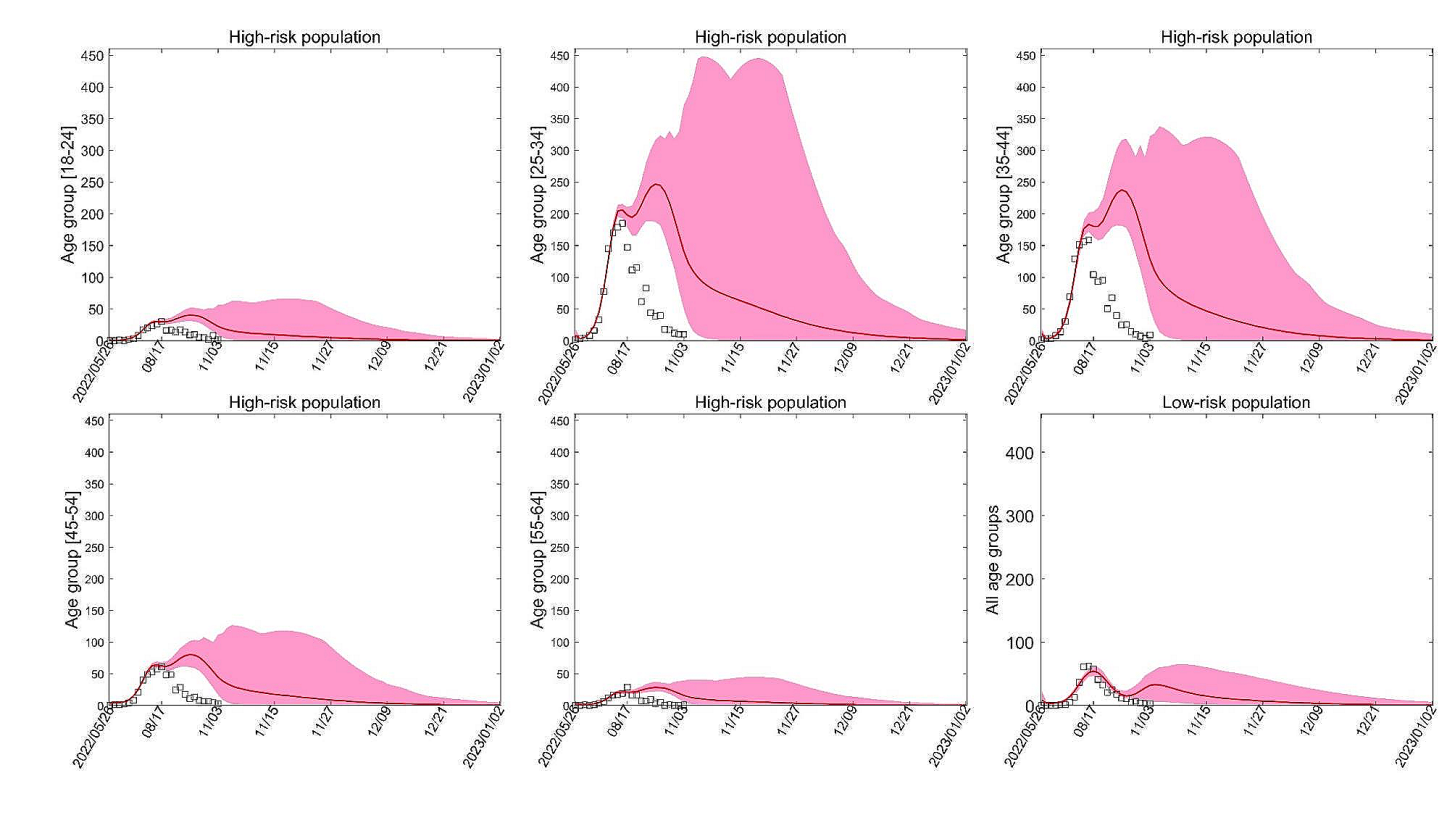
Numerical simulations for New York City in the absence of vaccination, i.e., ${\overline{\sigma}{} }_{\varvec{i}}^{\varvec{h}}=0,\hspace{0.25em}{\stackrel{\sim}{\varvec{\sigma }}}_{\varvec{i}}^{\varvec{h}}=0,\hspace{0.25em}{\overline{\sigma}{} }^{\varvec{l}}=0,\hspace{0.25em}{\stackrel{\sim}{\varvec{\sigma }}}^{\varvec{l}}=0$ ($1\le \varvec{i}\le 5$)

For various reasons, individuals in some areas may choose not to be vaccinated. To evaluate the impact of this scenario, a numerical simulation from mpox virus transmission model is presented in Fig. 2. The simulation indicates that in the absence of vaccination, the number of new infections in New York City will surge by approximately 385% and the transmission time will increase by about 350 %

**Fig. 3:**
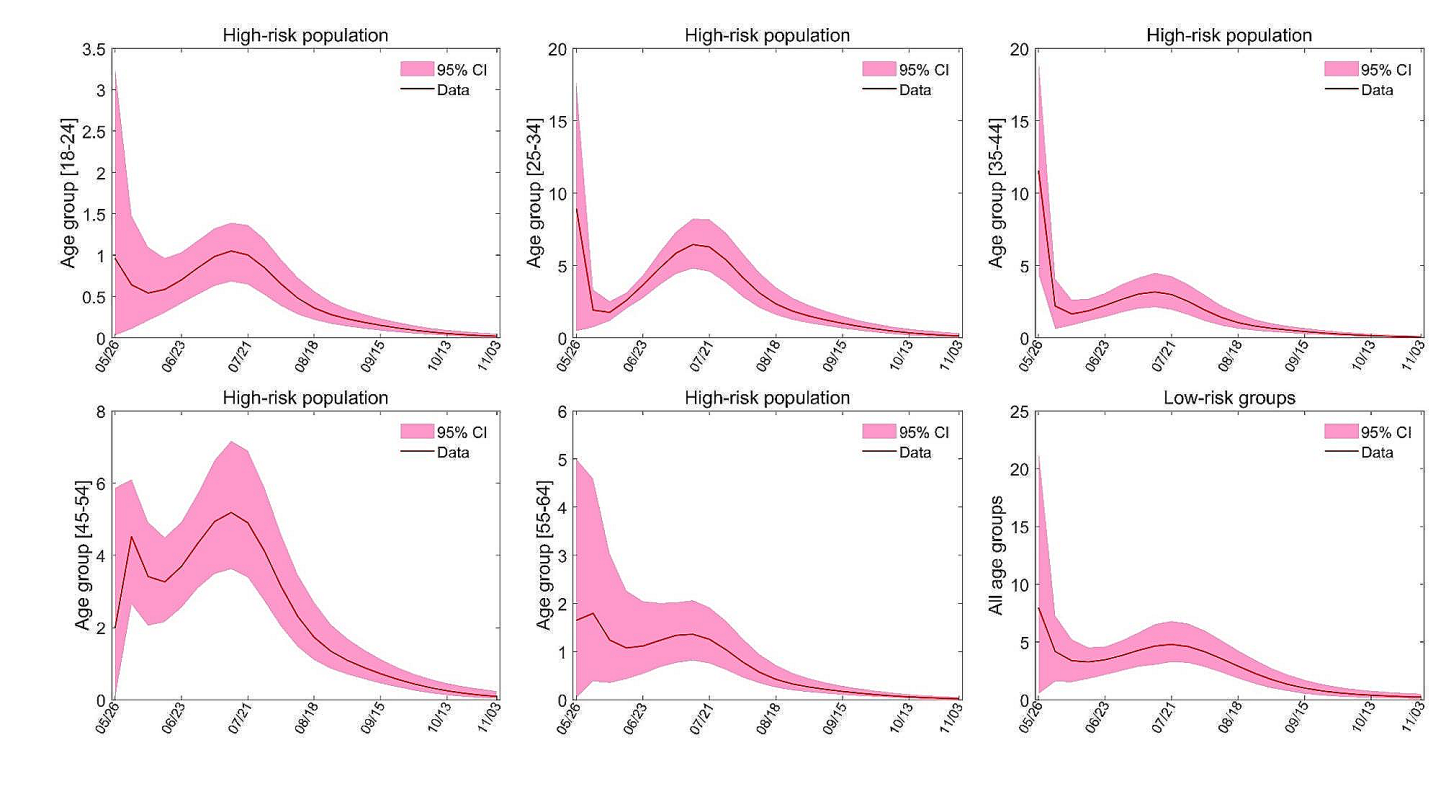
Numerical simulation results of vaccination strategy (1). (A)-(E) represents the number of new cases in different age groups of high-risk population. (F) shows the number of new cases in low-risk groups

**Fig. 4:**
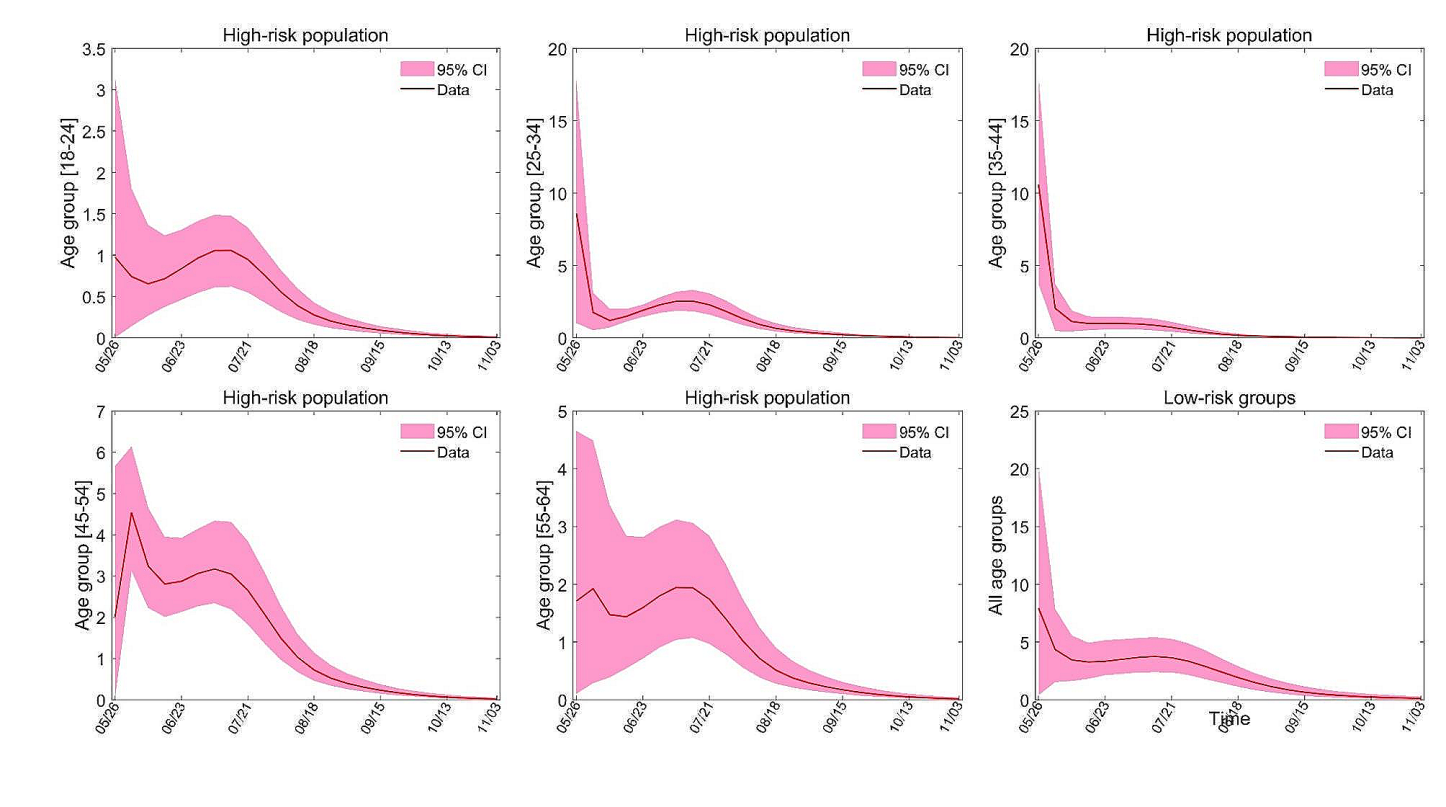
Numerical simulation results of vaccination strategy (2). (A)-(E) represents the number of new cases in different age groups of high-risk population. (F) shows the number of new cases in low-risk groups

In certain regions with considerable medical resources and attention paid to the mpox virus, policymakers may opt to administer prophylactic vaccinations before the exogenous virus infiltrates. In light of the data obtained from New York City, we made the assumption that inhabitants had received vaccinations prior to the outbreak’s onset. We evaluated two vaccination strategies: (1) complete vaccination before the epidemic according to the age-based vaccine allocation strategy in New York City (see Figure S4 in Supplementary Appendix), (2) complete vaccination before the beginning of the epidemic according to the sensitivity analysis (see Figure S10). The simulation of these two strategies is achieved by changing the initial value of the model (see Table 1). The numerical simulation results of both strategies are shown in Figs. 3 and 4, respectively. Based on the numerical results, vaccination under strategy (1) reduces the number of new infections by about 94.2% compared to the New York City vaccination program. For vaccination strategy (2), the number of new infections is reduced by 96% compared to the New York City vaccination program and decreased by 30.6% compared to the vaccination strategy (1).

**Table 1:** Initial values under several strategies. The scenario (i) corresponds to non-vaccination. The scenario (ii) represents that vaccination has been completed before the pandemic, in accordance with the current New York City policy on the distribution of vaccines for different age groups. The scenario (iii) represents that vaccination was completed prior to the outbreak based on sensitivity analysis. For comparison, the size of vaccinations in scenario (ii) and scenario (iii) is the same

Scenario (i)	Scenario (ii)	Scenario (iii)
${S}_{1}^{h}=16,000$	${S}_{1}^{h}=6254$	${S}_{1}^{h}=8616$
${\stackrel{\sim}{V}}_{1}^{h}=0$	${\stackrel{\sim}{V}}_{1}^{h}=0$	${\stackrel{\sim}{V}}_{1}^{h}=0$
${\overline{V}{}}_{1}^{h}=0$	${\overline{V}{}}_{1}^{h}=9746$	${\overline{V}{}}_{1}^{h}=7384$
${S}_{2}^{h}=45,953$	${S}_{2}^{h}=6160$	${S}_{2}^{h}=0$
${\stackrel{\sim}{V}}_{2}^{h}=0$	${\stackrel{\sim}{V}}_{2}^{h}=0$	${\stackrel{\sim}{V}}_{2}^{h}=0$
${\overline{V}{}}_{2}^{h}=0$	${\overline{V}{}}_{2}^{h}=39,793$	${\overline{V}{}}_{2}^{h}=45,953$
${S}_{3}^{h}=30,047$	${S}_{3}^{h}=5237$	${S}_{3}^{h}=0$
${\stackrel{\sim}{V}}_{3}^{h}=0$	${\stackrel{\sim}{V}}_{3}^{h}=0$	${\stackrel{\sim}{V}}_{3}^{h}=0$
${\overline{V}{}}_{3}^{h}=0$	${\overline{V}{}}_{3}^{h}=24,810$	${\overline{V}{}}_{3}^{h}=30,047$
${S}_{4}^{h}=1820$	${S}_{4}^{h}=1820$	${S}_{4}^{h}=1820$
${\stackrel{\sim}{V}}_{4}^{h}=16,380$	${\stackrel{\sim}{V}}_{4}^{h}=3872$	${\stackrel{\sim}{V}}_{4}^{h}=0$
${\overline{V}{}}_{4}^{h}=0$	${\overline{V}{}}_{4}^{h}=12,508$	${\overline{V}{}}_{4}^{h}=16,380$
${S}_{5}^{h}=1680$	${S}_{5}^{h}=1680$	${S}_{5}^{h}=1680$
${\stackrel{\sim}{V}}_{5}^{h}=15,120$	${\stackrel{\sim}{V}}_{5}^{h}=5677$	${\stackrel{\sim}{V}}_{5}^{h}=15,120$
${\overline{V}{}}_{5}^{h}=0$	${\overline{V}{}}_{5}^{h}=9443$	${\overline{V}{}}_{5}^{h}=0$

## Discussion

Mpox outbreaks are transmitted insidiously among men who have sex with men, with about 85% of cases resulting from high-risk behaviors. Furthermore, statistics from New York City display an age distribution in recent mpox cases, indicating a correlation between prevalence and age. Therefore, vaccination strategies that account for age heterogeneity can be more effective in reducing the number of new cases than age homogeneity. Furthermore, statistics from New York vaccination against smallpox is the only way for humans to acquire protection as there is no exclusive vaccine for mpox. However, the smallpox vaccine has a half-life of 92 years and even newly vaccinated people have only 80% protection against mpox virus. Neglecting the waning of smallpox vaccine protection would lead to an underestimation of the extent of mpox. Therefore, this study uses a mathematic model that takes into account high-risk and low-risk population, specific age structure, age exposure structure, and vaccine failure rate to assess and compare smallpox vaccination strategies. We use the MCMC method to estimate unknown parameters for the age-structured model. Then, we derive effective reproduction numbers that describe the transmissibility of mpox when combined with prevention and control interventions (vaccination, isolation, etc.). Effective reproduction numbers in New York City are calculated based on the parameters obtained by fitting, and the results shows that mpox would not break out in the general population. In particular, our results also indicate that this mpox outbreak in New York City may be caused by Pride Festival, which has increased the contacts among high-risk population. Additionally, based on the sensitivity analysis results for New York City, we find that prophylactic vaccination is the most effective for high-risk group aged 34–45. The individuals in this age group may have specific risk factors because they are in a relatively optimal physical, psychological, and economic situation, resulting in high levels of sexual activity but have not been vaccinated against smallpox. This recommendation is valid since various age groups respond differently to preventive vaccination in terms of reducing the number of new occurrences. The sensitivity of age groups from high to low is as follows: 35–44, (45–54 or 25–34), 18–24, 64+, which is obtained from the sensitivity analysis. This result is also reasonable when taking into account physical, economic status and vaccination history. It should be noted that the sensitivity of vaccination rates tends to decrease as the epidemic progresses. This means that the timing of vaccination is important in reducing mpox transmission. Further, according to the data from New York City, three vaccination scenarios are considered to assess the impact of different strategies on mpox transmission. The first scenario is characterized by the fact that no one is vaccinated during an epidemic. The second scenario features vaccination prior to mpox virus transmission based on the current vaccination schedule in New York City (see Table S2 or Figure S4 in Supplementary Appendix). The third scenario is characterized by prophylactic vaccination based on the results of a sensitivity analysis. Therefore, we conclude that prophylactic vaccination in high-risk population in increasing order of priority for age groups 35–44, 45–54, 25–34, 18–24, 64+ is an effective strategy. In addition, based on the fact that mpox virus evolves slowly, the results of Scenario 2 show that New York City will not have a mpox epidemic on the scale of 2022 in recent years, even if another Pride festival-like event is held.

**Fig. 5:**
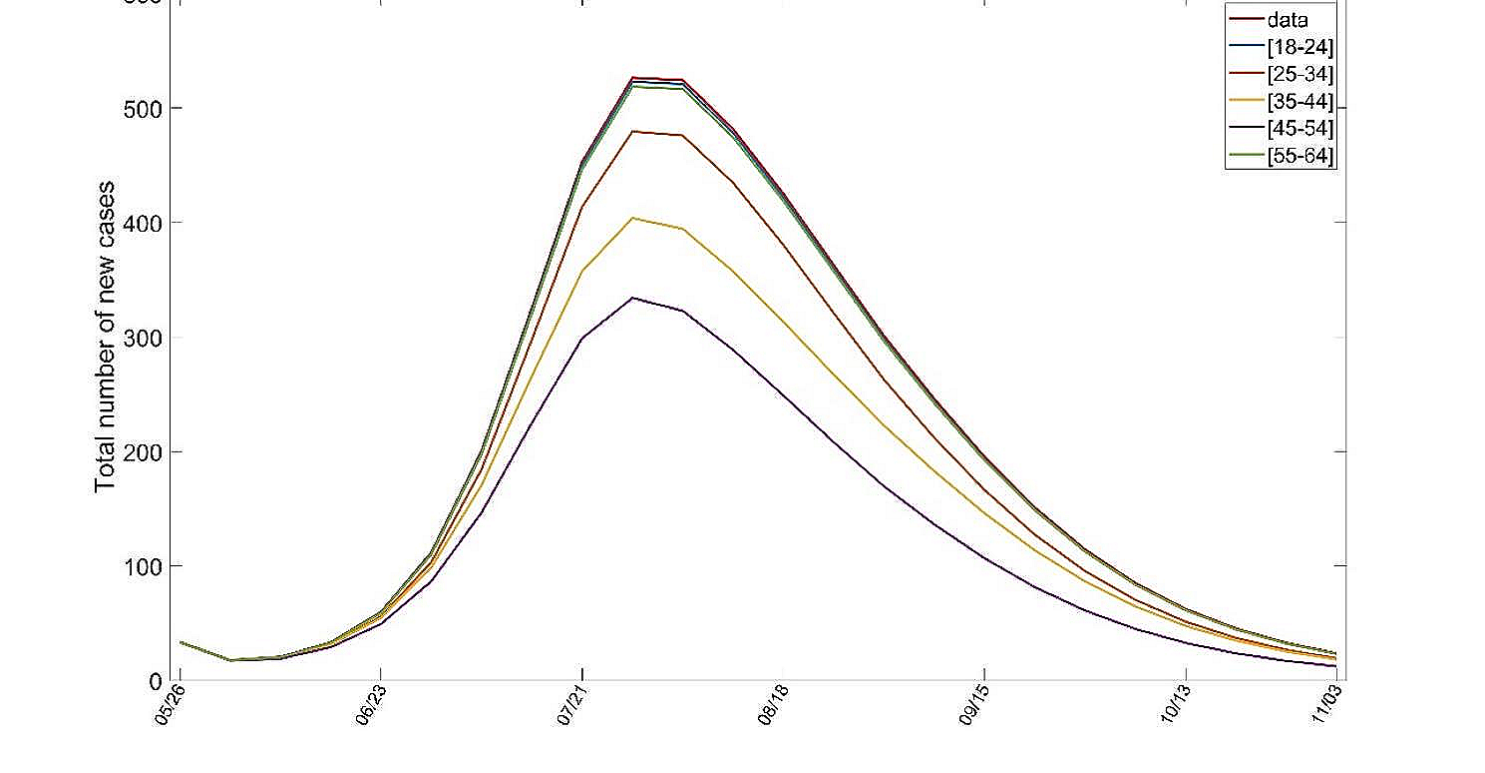
Numerical simulation results on the effect of reducing the transmission rate by age group on the total number of new cases. The curves represent the decrease in the total number of new cases due to a 10% reduction in transmission rates in each age group

We have investigated the impact of vaccination strategies on the spread of mpox virus. In fact, reducing the transmission rate is also a way to control mpox epidemic. We simulated the total number of new cases by reducing the transmission rate of each age group to explore which age group in the high-risk population has the most effective transmission rate for disease control. Numerical results show that the effect of reducing the transmission rate of 45–55 years old is the most effective (see Fig. 5).

## Conclusions

The mpox outbreak in New York City may be linked to the Pride event. However, with current vaccine coverage, there will be no more large-scale outbreaks of mpox, even if there is another similar activity. For areas with limited vaccines, priority is given to high-risk groups in the age group 34–45 years as soon as possible.

## Electronic supplementary material

Below is the link to the electronic supplementary material.

Supplementary Material 1

## Data Availability

Data and materials were obtained from public domain resources as cited in the paper.
