# Supplementary material for: Assessing vaccine strategies for mpox outbreak in New York City using an age-structure model
Source: BMC Infect Dis. 2024 Sep 30;24:1078. doi: 10.1186/s12879-024-09551-2 (PMC11441002; doi:10.1186/s12879-024-09551-2)
Supplement: Supplementary file 1 — Supplementary Material 1 [file 12879_2024_9551_MOESM1_ESM.pdf]

Mathematical Details: mpox Virus Transmission Model Approach

Zixiao Xiong, Ling Xue, Xuezhi Li, Yanfen Zhang

We construct a compartmental mpox virus transmission model that takes into account the division of risk groups, the distribution of age, the dynamics of vaccination, and the weakening of vaccine efficacy. Here, we present modeling details and describe the computational and mathematical methods used to obtain parameter estimates, as well as the results presented in the text.

The rest of the documents is organized as follows. In Section 1, we introduce the research status and modeling details of mpox virus transmission model. In Section 2, we give weekly new mpox cases and vaccination doses from May 19 to November 3, 2022. Section 3 discusses the initial state and parameter range of the model and the fitting results. Section 4 gives the derivation of effective regeneration numbers and the results in New York City. Section 5 presents a sensitivity analysis of vaccination to mpox outbreaks.

Contents

1 Mpox Virus Transmission Model Structure . . . **1**
- 1.1 Disease Progression . . . 2
- 1.2 Population Division . . . 2
- 1.3 Some Assumptions . . . 3
- 1.4 Mpox Virus Transmission Model . . . 3

2 Surveillance Data . . . **7**

3 Model Calibration . . . **8**
- 3.1 Population Parameters . . . 8
- 3.2 Model Parameters . . . 10
  - 3.2.1 Disease Progression Parameters . . . 10
  - 3.2.2 Disease Transmission Parameters . . . 11
  - 3.2.3 Vaccination Parameters . . . 11
- 3.3 Fitting Result . . . 12

4 Effective Reproduction Number . . . **12**
- 4.1 Theoretical Derivation . . . 12
- 4.2 Effective Reproduction Number For New York City . . . 17

5 Sensitivity Analysis . . . **17**

1. Mpox Virus Transmission Model Structure

Mathematical models have become an important tool for infectious disease prevention, control, and prediction [1, 2]. Many mathematical models of mpox have been proposed [3–9]. Since previous outbreaks

of Mpox were mainly primary cases, many models of two groups (animals and humans) were established [3–7]. In recent years, most of the mathematical models on mpox only consider the spread among humans, because the main route of mpox transmission has changed to human-to-human [8, 9]. Additionally, many models on COVID-19 and tuberculosis have taken into account age structure and vaccination strategies, which are often neglected in mpox [10, 11]. Many results suggest that effective vaccine distribution strategies can effectively reduce economic costs and new cases when vaccines are limited [10, 11]. Therefore, for mpox, vaccine allocation decisions are crucial.

From May 7, 2022, the mpox epidemic began to spread globally, and the number of mpox cases increased rapidly [12, 13]. Notably, currently 98% of mpox patients are currently male, and more than 95% of them are men who have sex with men [14]. Therefore, we considered gay and bisexual individuals as high-risk population. The mpox is mainly transmitted through droplets, direct contact with body fluids or lesion material, and indirect contact with lesion material via contaminated fomites in low-risk population. In fact, published data show a clear age distribution of mpox cases in high-risk populations. This means that the risk of transmission of the mpox virus varies in different age groups. Additionally, we ignored the age distribution of low-risk population due to the small number of cases. Currently, the commonly used smallpox vaccine provides 85 percent effective in preventing the mpox virus invasion [15]. However, the protection of smallpox vaccine will gradually wane [16].

1.1. *Disease Progression*

The progression of mpox infection is divided into three stages: the incubation period, the rash period, and the recovery period as shown in Figure S1. The incubation period is then split into two further stages, namely, exposure period and prodromal period. Once infected with the mpox virus, individuals first experience an asymptomatic incubation period that can last for 5 to 21 days [15]. Symptoms such as fever, severe headache, and swollen lymph nodes occur in the prodromal phase about 1-4 days before the rash appears [15]. In the rash stage, symptoms (approximately 2-4 weeks) are macules (lesions with a flat base) papules (slightly raised, firm lesions), vesicles (lesions filled with clear fluid), and pustules (lesions filled with yellowish fluid), dry crusts [15]. Most people recover spontaneously and do not have a relapse [15]. It is worth noting that patients in the prodromal phase have no symptoms but are infectious [17].

1.2. *Population Division*

The population segmentation of the model is shown in Figure S2. We classify population as high-risk and low-risk, and let N represent the size in both populations, i.e., $N = N^h + N^l$ where N^h and N^l denote the size of high-risk and low-risk population, respectively. We further divide the high-risk population into n age-groups as $[x_1, x_2), \cdots, [x_i, x_{i+1}), \cdots\ [x_n, x_{n+1})$, where $i, n \in \mathbb{N}$ and $x_i \in [0\ \infty]$ indicates age. Then, in the ith age group $[x_i, x_{i+1})$, the high-risk population is classified into seven compartments: susceptible (S_i^h), previously vaccinated ($\tilde{V}_i^h$), currently vaccinated ($\bar{V}_i^h$) exposed (E_i^h), prodromal (P_i^h), infected (I_i^h) and recovered (R_i^h), where $S_i^h + \tilde{V}_i^h + \bar{V}_i^h + E_i^h + P_i^h + I_i^h + R_i^h = N_i^h$, which is the size of high-risk population in the ith age group, and $\sum_{i=1}^n N_i^h = N^h$. Moveover, the low-risk population was divided into seven compartments: susceptible (S^l), previously vaccinated ($\tilde{V}^l$), currently vaccinated ($\bar{V}^l$), exposed (E^l), prodromal (P^l), infected (I^l), and recovered (R^l) without distinction of age and $S^l + \tilde{V}^l + \bar{V}^l + E^l + P^l + I^l + R^l = N^l$.

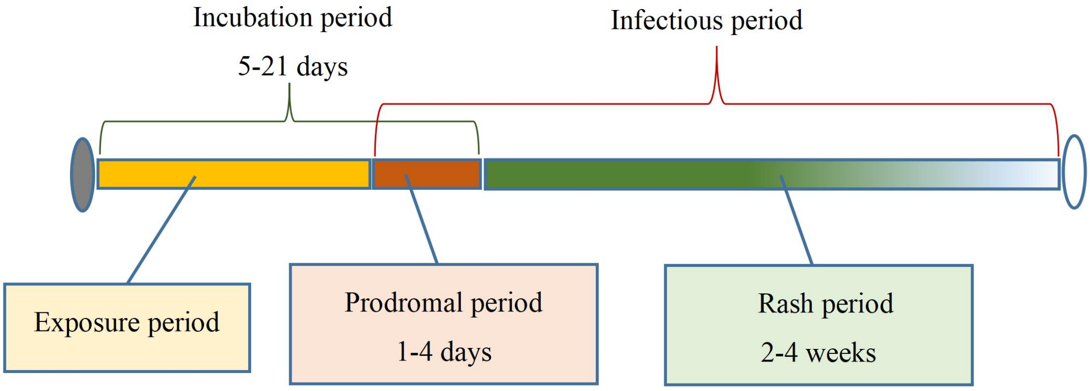

Figure S1: Diagram of the progression of mpox

1.3. Some Assumptions

We make the following assumptions: (i) duration of an epidemic is short compared to the humans lifespan meaning we ignore the birth rate, natural death rate and transfer rates from age-group i to $i+1$. (ii) mpox is a self-limiting disease with mortality rate only about 0.028% [18]. Thus, we ignore the deaths caused by mpox.

1.4. Mpox Virus Transmission Model

The spread of mpox among various compartments and age groups in high-risk and low-risk populations is depicted in flowchart in Figure S3. Susceptible individuals in the i-th age-group of high-risk population can be infected by prodromal and infected individuals of all age-groups in high-risk population through high-risk behavior, and transfer to exposed compartment at the rate

$$f_i^{hh} = \alpha_i^h \sum_{j=1}^{n} \beta^h c_{ij}^h \left(\frac{\epsilon_j^h P_j^h + I_j^h}{N_j^h}\right), \tag{1}$$

where β^h is the probability of contacting an infection through high-risk behaviors with an infected individual, α_i^{hh} is the average contact number of the i-th age group of the high-risk population to the high-risk population, c_{ij}^h is the contact probability of the i-th age-group to the j-th age-group within high-risk population. The infectivity during prodromal period is weakened compared to that during the infectious phase, with ϵ_j^h representing the level of weakening in j-th age-group of high-risk population. $(P_j^h + I_j^h)/N_j^h$ is the probability that an individual encounter an prodromal or infectious period individuals of age-group j in high-risk population. Susceptible individuals in age group i of high-risk population can also be infected by prodromal and infected individuals in low-risk population through low-risk behaviors and transfer to exposed compartment at the rate

$$f_{hl} = \alpha_i^{hl} \beta^l c_i^{hl} \left(\frac{\epsilon^l P^l + I^l}{N^l}\right), \tag{2}$$

where β^l is the probability of engaging in low-risk behaviors with an infected individuals and being infected, α_i^{hl} is the average contact number of the i-th age group of the high-risk population to the low-risk

population, c_i^{hl} is the contact probability of the i-th age-group in high-risk population to the low-risk population. Similarly, ϵ^l represents the weakening degree of the prodromal phase compared to the infection phase in low-risk populations. $(P^l + I^l)/N^l$ is the probability that an individual encounters prodromal or infectious individuals period in low-risk population. Susceptible individuals in age group i of high-risk population can enter currently vaccinated compartment ($\tilde{V}$) through vaccination at the rate $\bar{\sigma}_i^h$. Previously vaccinated individuals are effectively re-vaccinated at a rate of $\tilde{\sigma}_i^h$. Considering that vaccination does not provide 100% protection, vaccinated individuals (whether previously vaccinated or currently, i.e., $\tilde{V}_i^h$ or $\bar{V}_i^h$) may enter the compartment of infected. $\tilde{\delta}_i^h$ and $\bar{\delta}_i^h$ are the relative susceptibility of vaccinated individuals in $\tilde{V}_i^h$ and $\bar{V}_i^h$ compared to susceptible compartment in high-risk populations. Therefore, individuals in $\tilde{V}_i^h$ and $\bar{V}_i^h$ enter the compartment of the infected at rates $\left(\tilde{\delta}_i^h(f_{hh} + f_{lh}) \text{ and } \bar{\delta}_i^h(f_{hh} + f_{lh})\right)$, respectively. Exposed individuals in the i-th age group of high-risk population transfer to the prodromal compartment of age-group i in high-risk population at the rate η_i^h. Further, the prodromal individuals in the i-th age-group of high-risk population transfer to the infected compartment of the age-group i in high-risk population at the rate μ_i^h. Finally, infected individuals in the i-th age-group in high-risk population recover at the rate γ_i^h to the high-risk recovered compartment of the same age group. For low-risk population, the mpox virus spreads in a similar way as high-risk population. The rate at which susceptible individuals in low-risk populations infected by prodromal and infectious individuals of all age groups in high-risk populations is given by the following equation

$$f_{lh} = \sum_{j=1}^{n} \beta^l c_j^{lh} \left(\frac{\epsilon_j^h P_j^h + I_j^h}{N_j^h}\right), \tag{3}$$

where c_j^{lh} is the contact probability of low-risk population to the j-th age-group high-risk population. Similarly, the rate at which low-risk individuals are infected by low-risk prodromal and infectious phases individuals is described by Equation (4) as

$$f_{ll} = \beta^l c_0^l \left(\frac{\epsilon^l P^l + I^l}{N^l}\right), \tag{4}$$

where c_0^l is the number of contacts of humans in low-risk population. $\tilde{\delta}^l$ and $\bar{\delta}^l$ are the relative susceptibility of vaccinated individuals in $\tilde{V}^l$ and $\bar{V}^l$ compared to susceptible compartment in low-risk populations. η^l is the transfer rate from exposure to the prodromal stage in low-risk population. μ^l indicates the transfer rate from prodromal to rash stage in low-risk population. γ^l is the recovery rate of low-risk population. The corresponding mathematical model is described in Equation (5).

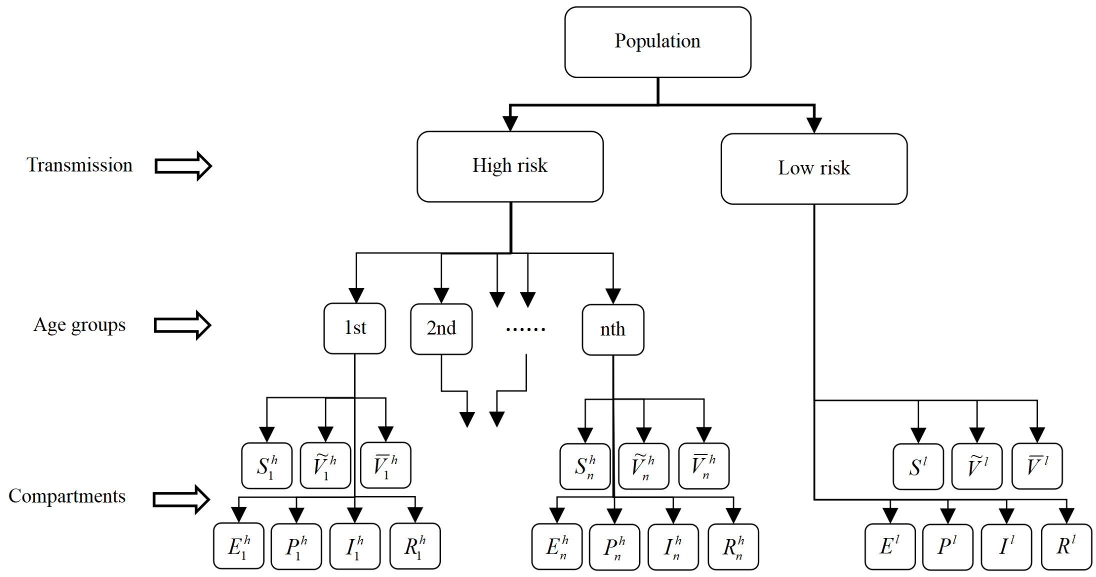

Figure S2: Tree of population classification. The population under study is divided into high-risk and low-risk. Because patients in high-risk population showed a clear age distribution, we divide high-risk population into n groups according to age. Further, based on physical state, we classify individuals into six compartments, namely, susceptible, previously vaccinated, currently vaccinated, exposed, prodromal, infected, and recovered.

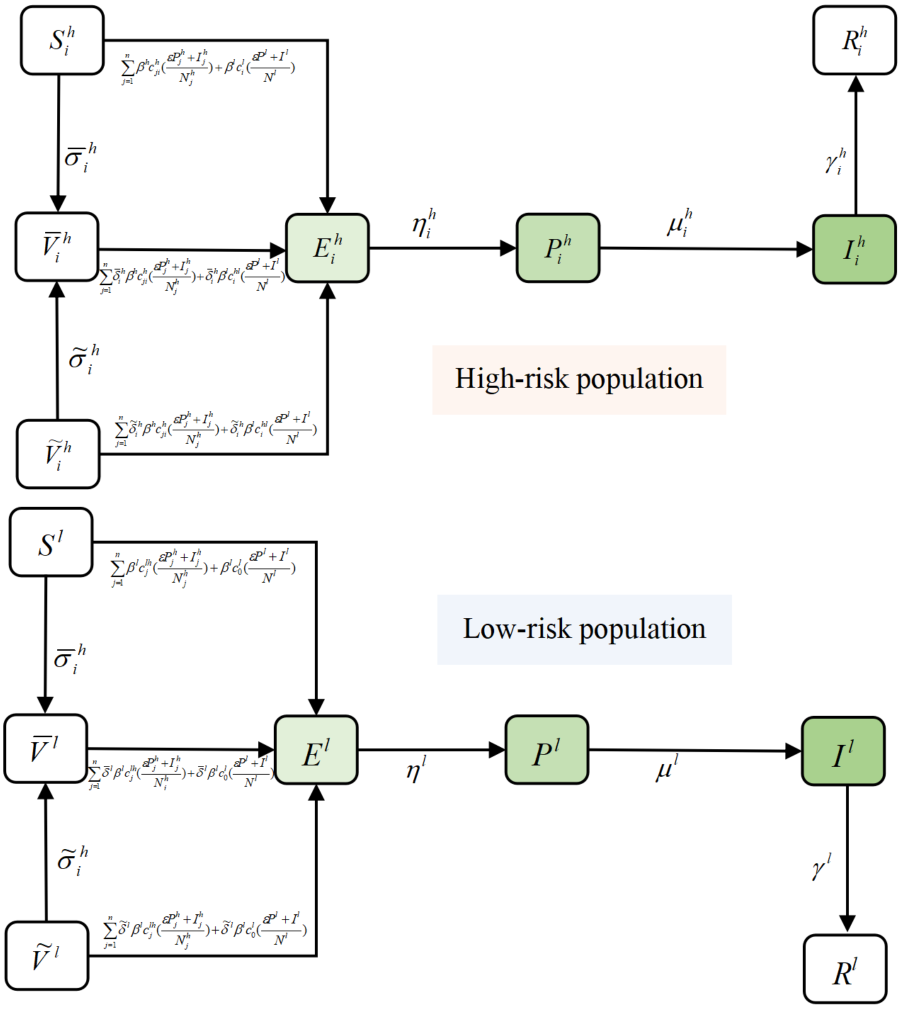

Figure S3: Schematic diagram of the mathematical model.

$$
\begin{cases}
\frac{dS_i^h}{dt} = -\alpha_i^{hh}\beta^h \sum_{j=1}^{n} c_{ij}^h (\frac{\epsilon_j^h P_j^h + I_j^h}{N_j^h}) S_i^h - \alpha_i^{hl}\beta^l c_i^{hl} (\frac{\epsilon^l P^l + I^l}{N^l}) S_i^h - \bar{\sigma}_i^h S_i^h; \\
\frac{d\tilde{V}_i^h}{dt} = -\tilde{\delta}_i^h \alpha_i^{hh}\beta^h \sum_{j=1}^{n} c_{ij}^h (\frac{\epsilon_j^h P_j^h + I_j^h}{N_j^h}) \tilde{V}_i^h - \tilde{\delta}_i^h \alpha_i^{hl}\beta^l c_i^{hl} (\frac{\epsilon^l P^l + I^l}{N^l}) \tilde{V}_i^h - \tilde{\sigma}_i^h \tilde{V}_i^h; \\
\frac{d\bar{V}_i^h}{dt} = \bar{\sigma}_i^h S_i^h + \tilde{\sigma}_i^h \tilde{V}_i^h - \bar{\delta}_i^h \alpha_i^{hh}\beta^h \sum_{j=1}^{n} c_{ij}^h (\frac{\epsilon_j^h P_j^h + I_j^h}{N_j^h}) \bar{V}_i^h - \bar{\delta}_i^h \alpha_i^{hl}\beta^l c_i^{hl} (\frac{\epsilon^l P^l + I^l}{N^l}) \bar{V}_i^h; \\
\frac{dE_i^h}{dt} = \alpha_i^{hh}\beta^h \sum_{j=1}^{n} c_{ij}^h (\frac{\epsilon_j^h P_j^h + I_j^h}{N_j^h})(S_i^h + \tilde{\delta}_i^h \tilde{V}_i^h + \bar{\delta}_i^h \bar{V}_i^h) + \alpha_i^{hl}\beta^l c_i^{hl} (\frac{\epsilon^l P^l + I^l}{N^l})(S_i^h + \tilde{\delta}_i^h \tilde{V}_i^h + \bar{\delta}_i^h \bar{V}_i^h) - \eta_i^h E_i^h; \\
\frac{dP_i^h}{dt} = \eta_i^h E_i^h - \mu_i^h P_i^h \\
\frac{dI_i^h}{dt} = \mu_i^h P_i^h - \gamma_i^h I_i^h; \\
\frac{dR_i^h}{dt} = \gamma_i^h I_i^h; \\
\frac{dS^l}{dt} = -\alpha^{lh}\beta^l \sum_{j=1}^{n} c_j^{lh} (\frac{\epsilon_j^h P_j^h + I_j^h}{N_j^h}) S^l - \alpha^{ll}\beta^l c_0^l (\frac{\epsilon^l P^l + I^l}{N^l}) S^l - \bar{\sigma}^l S^l; \\
\frac{d\tilde{V}^l}{dt} = -\tilde{\delta}^l \alpha^{lh}\beta^l \sum_{j=1}^{n} c_j^{lh} (\frac{\epsilon_j^h P_j^h + I_j^h}{N_j^h}) \tilde{V}^l - \tilde{\delta}^l \alpha^{ll}\beta^l c_0^l (\frac{\epsilon^l P^l + I^l}{N^l}) \tilde{V}^l - \tilde{\sigma}^l \tilde{V}^l; \\
\frac{d\bar{V}^l}{dt} = \bar{\sigma}^l S^l + \tilde{\sigma}^l \tilde{V}^l - \bar{\delta}^l \alpha^{lh}\beta^l \sum_{j=1}^{n} c_j^{lh} (\frac{\epsilon_j^h P_j^h + I_j^h}{N_j^h}) \bar{V}^l - \bar{\delta}^l \alpha^{ll}\beta^l c_0^l (\frac{\epsilon^l P^l + I^l}{N^l}) \bar{V}^l; \\
\frac{dE^l}{dt} = \alpha^{lh}\beta^l \sum_{j=1}^{n} c_j^{lh} (\frac{\epsilon_j^h P_j^h + I_j^h}{N_j^h})(S^l + \tilde{\delta}^l \tilde{V}^l + \bar{\delta}^l \bar{V}^l) + \alpha^{ll}\beta^l c_0^l (\frac{\epsilon^l P^l + I^l}{N^l})(S^l + \tilde{\delta}^l \tilde{V}^l + \bar{\delta}^l \bar{V}^l) - \eta^l E^l; \\
\frac{dP^l}{dt} = \eta^l E^l - \mu^l P^l \\
\frac{dI^l}{dt} = \mu^l P^l - \gamma^l I^l; \\
\frac{dR^l}{dt} = \gamma^l I^l;
\end{cases}
\quad (5)
$$

2. Surveillance Data

The first case of mpox in New York City occurred since May 19, 2022. As of November 3 of the same year, the mpox outbreak in New York City almost ended [19]. The New York City Health provides ages of individuals infected with the mpox virus and weekly incidence since May 19. After performing the data analysis, we have computed the new cases for the five age groups of high-risk and low-risk population which are listed in Table S1. The data indicate that high-risk sexual behaviour was a predominant factor contributing to the majority of mpox cases in non-endemic areas [14]. Referring to WHO data, we give an estimate of the proportion of high-risk sexual behavior in the number of mpox cases in New York City in Table S1. In addition, the weekly vaccination data from May 19 to November 3 is shown in Table S2

[19]. For later evaluation, we also summarized the total number of vaccinations for each group as shown in Figure S4.

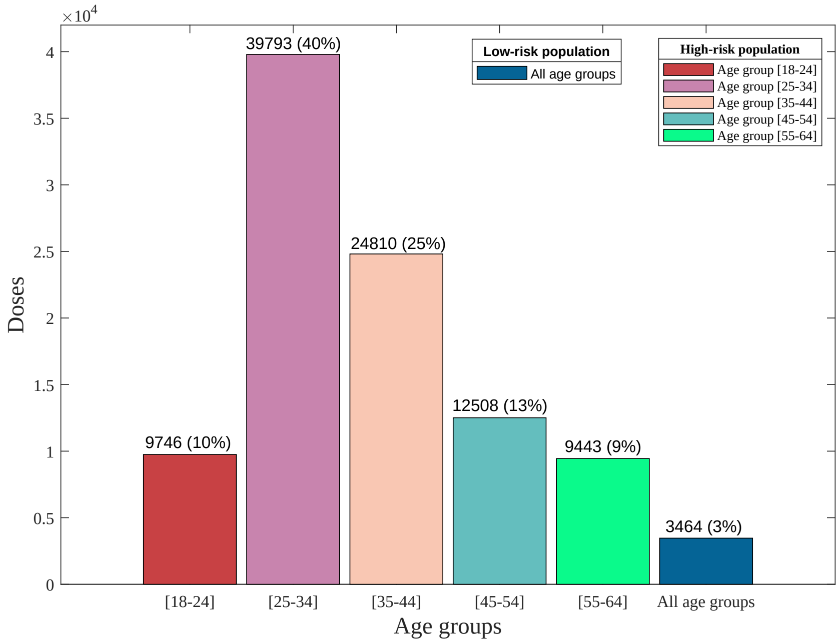

Figure S4: The vaccine distribution for each age group of high-risk population and low-risk population in New York City from May 19 to November 3.

3. Model Calibration

In this section, we determine the parameter range based on literature review, expert opinion, and fit mpox data published by New York City Health using the Markov Chain Monte Carlo algorithm (MCMC).

3.1. *Population Parameters*

The citizens of New York City are divided into high-risk and low-risk group population. Further, the high-risk population are divided into the following five age-groups: 18-24 years old, 25-34 years old, 35-44 years old, 45-54 years old, and 55-64 years old. Next, the initial values and the parameters of the Model (5) are given. According to the results in [20], the population size of New York City in 2022 is about $N =$ 8930005. High-risk population are those who may have sex with men (gay and bisexual men). The sizes of each age group of high-risk population in New York City are $N_1^h = 16001$, $N_2^h = 45962$, $N_3^h = 30054$, $N_4^h = 18210$ and $N_5^h = 16802$, respectively [21]. The rest of the citizens in New York City are classified as low-risk population ($N^l = N - N^h = 8802976$). Notably, the smallpox vaccine can reduce the risk of contacting mpox virus. We assume that smallpox vaccination in the U.S. was completely ended in 1978, and 90 percent of the population aged over 44 is protected [23]. Meanwhile, we assume that people born after 1978 have not been vaccinated against smallpox. Therefore, we take $S_1^h = 16000$, $\tilde{V}_1^h = 0$, $\bar{V}_1^h = 0$, $S_2^h = 45953$, $\tilde{V}_2^h = 0$, $\bar{V}_2^h = 0$, $S_3^h = 30047$, $\tilde{V}_3^h = 0$, $\bar{V}_3^h = 0$, $S_4^h = 1820$, $\tilde{V}_4^h = 16380$, $\bar{V}_4^h = 0$ and $S_5^h = 1680$, $\tilde{V}_5^h = 15120$, $\bar{V}_5^h = 0$. The initial number of individuals during exposure period (E_i^h, $0 \leq i \leq 5$) and the prodromal period (P_i^h, $0 \leq i \leq 5$) that cannot be counted are estimated from Model (5) by MCMC.

Table S1: NCC* of mpox in New York City in different population and age groups [19]

Time	2022/05/26	2022/06/02	2022/06/09	2022/06/16	2022/06/23	2022/06/30	2022/07 /07	2022/07 /14	2022/07 /21	2022/07 /21	2022/08/04	2022/08/11	2022/08/18	2022/08/25	2022/09/01	2022/09/08	2022/09/15	2022/09/22	2022/09/29	2022/10/06	2022/10/13	2022/10/20	2022/10/27	2022/11/03
									NCC*															
Total NCC*	4	7	10	18	38	78	186	356	442	489	508	429	325	324	177	227	142	97	104	51	46	31	29	27
Proportion of HRP*	1	1	1	1	0.97	0.98	0.97	0.96	0.91	0.87	0.87	0.86	0.87	0.9	0.88	0.89	0.87	0.88	0.9	0.9	0.86	0.87	0.9	0.9
									HRP*															
Age group [18-24]	0	0	1	0	2	3	8	17	20	23	26	30	16	16	14	17	14	9	10	5	5	2	8	2
Age group [25-34]	2	3	4	8	16	33	77	145	170	179	185	147	111	115	61	83	44	39	40	18	17	12	10	10
Age group [35-44]	2	3	3	8	14	30	69	129	151	156	159	104	93	95	50	68	40	24	25	14	10	7	4	9
Age group [45-54]	0	1	1	2	4	8	21	40	49	53	57	61	48	49	24	29	18	11	14	8	6	6	5	3
Age group [55-64]	0	0	1	0	1	3	6	12	16	17	19	29	16	16	7	6	9	3	5	0	2	0	0	1
									LRP*															
All age groups	0	0	0	0	1	1	5	13	36	61	62	58	41	32	20	25	18	12	10	5	6	4	3	3

NCC: Newly confirmed cases, HRP: High-risk population, LRP: Low-risk population.

Table S2: NVC* of mpox in New York City in different population and age groups [19]

Time	2022/05/26	2022/06/02	2022/06/09	2022/06/16	2022/06/23	2022/06/30	2022/07 /07	2022/07 /14	2022/07 /21	2022/07 /21	2022/08/04	2022/08/11	2022/08/18	2022/08/25	2022/09/01	2022/09/08	2022/09/15	2022/09/22	2022/09/29	2022/10/06	2022/10/13	2022/10/20	2022/10/27	2022/11/03
									NVC*															
Total NVC*	2	8	10	8	24	877	103	2928	7496	8033	12960	18291	19712	6531	6629	4433	3049	2582	1867	1361	1114	943	803	490
									HRP*															
Age group [18-24]	0	1	1	1	2	86	10	286	732	785	1266	1787	1926	638	648	433	298	252	182	133	109	92	78	44
Age group [25-34]	1	3	4	3	10	350	41	1168	2990	3204	5169	7296	7863	2605	2644	1768	1216	1030	745	543	444	376	320	195
Age group [35-44]	1	2	3	2	6	218	25	728	1864	1998	3223	4549	4902	1624	1649	1102	758	642	464	338	277	235	200	126
Age group [45-54]	0	1	1	1	3	110	13	367	940	1007	1625	2293	2471	819	831	556	382	324	234	171	140	118	101	63
Age group [55-64]	0	1	1	1	2	83	10	277	710	760	1227	1731	1866	618	627	420	289	244	177	129	105	89	76	46
									LRP*															
All age groups	0	0	0	0	1	30	4	102	260	279	450	635	684	227	230	154	106	90	65	47	39	33	28	16

NVC: Newly vaccinated cases, HRP: High-risk population, LRP: Low-risk population.

Meanwhile, in non-endemic area, we assume that the number of recovered individuals is zero, i.e., $R_i^h = 0$ where $0 \leq i \leq 5$. For low-risk population, the number of vaccinated individuals can be derived using the NYC age pyramid, that is, $\tilde{V}^l = 3122600 * 0.9 - \sum_{i=1}^{5} \tilde{V}_i^h = 2778840$ [20]. Similar to the high-risk population, the initial number of the exposed and prodromal humans in the low-risk population are estimated from the Model (5) by MCMC. We also consider that the number of recovered individuals in the low-risk population is zero, i.e., $R^l = 0$. Then, $S^l = N - N^h - V^l - E^l - P^l - I^l = 6024124$. The initial infected individuals can be obtained from Table S1, i.e., $I_1^h = 1$, $I_2^h = 2$, $I_3^h = 2$, $I_4^h = 0$, $I_5^h = 0$, $I^l = 0$. The final Population Parameters are shown in Table S3.

3.2. Model Parameters

We divide the model parameters into disease progression parameters, disease transmission parameters, and vaccination parameters. We list all the parameters in Table S4 and Table S5 .

3.2.1. Disease Progression Parameters

The incubation period of mpox virus in humans is 9 days and 13 days for high-risk and low-risk transmissions, respectively [24–26]. The transition rate of individuals in the prodromal and rash period are estimated from the Model (5) using the data within the ranges of $[7/4, 7/1]$ and $[7/28, 7/14]$, respectively. The experiment shows that the half-life of protection provided by the smallpox vaccine is 92 years [27]. For those who were vaccinated against smallpox before 1978, the reduction in protection rate satisfies the following equation

$$[Protection\ rate] = (1/2)^{\left(\frac{2022-[Vaccination\ date]}{92}\right)}. \tag{6}$$

We assume the vaccination date of 45+ individuals is their birth date. According to the Equation (6), vaccine

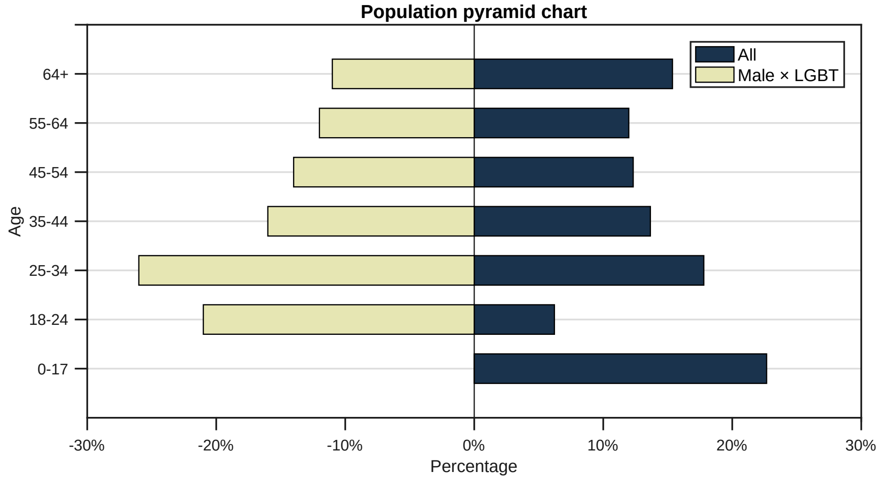

Figure S5: On the left is the age distribution of LGBT+ (Lesbian, Gay, Bisexual, Transgender) of man in New York City. On the right is the age distribution of the all New York City people.

effectiveness (85%) and chart of age distribution(Figure S5), we can get that the protection rates of smallpox vaccine in 45-54 and 55-64 years old are 0.59 and 0.55, respectively. Therefore, $\tilde{\delta}_4^h = 1 - 0.59 = 0.41$, $\tilde{\delta}_5^h = 1 - 0.55 = 0.45$. For people younger than 44 years old, $\tilde{\delta}_1^h = \tilde{\delta}_2^h = \tilde{\delta}_3^h = 1$. The protection rate for low-risk population is 0.31, which is the average of the protection rates for all the people in New York City. Thus, $\tilde{\delta}^l = 1 - 0.31 = 0.69$. The failure rate of vaccine protection is 0.15 in the newly vaccinated population

regardless of high or low risk, i.e., $\bar{\delta}_i^h = \bar{\delta}^l = 0.15,\ 0 \leq i \leq 5$. The probability of transmission during prodromal phase is $\epsilon \in [0,\ 0.5]$ times of that during infectious phase and estimated by the Model (5). The heterogeneities among individuals are ignored in the parameters estimation. Therefore, we have $\gamma^l = \gamma_i^h$, $\mu^l = \mu_i^h$, $\eta^l = \eta_i^h$, $\delta^l = \delta_i^h$, $\epsilon^l = \epsilon_i^h$, for $0 \leq i \leq 5$.

3.2.2. *Disease Transmission Parameters*

The $\beta_i^h \in [0,1]$, $(i = 1,\ 2, \cdots 5)$ and $\beta^l \in [0,1]$ are the probabilities of being infected due to high-risk and low-risk behaviors, respectively, which are estimated from Model (5). In addition, we infer that the daily new cases of mpox are closely related to behavior change and vaccination within high-risk population. We therefore assume that the average contacts number within high-risk population is time-dependent. Without loss of generality, we take the daily number of contacts in age group [45-54] from May 19 to November 3 as the baseline curve, which is fitted by a spline function based on the data. Further, we assume that the daily number of contacts in age groups [18-24], [25-34], [35-44] and [55-64] of the high-risk population is a multiple of age group [45-54], i.e., $\alpha_i^{hh} = m_i \alpha_4^{hh}(t),\ i = 1, 2, 3, 5$. The change of contacts over time is shown in Figure S9. We find that the average contact number in different age groups reaches the maximum between June 23 and June 30. It should be noted that on June 26, New York City hosted a Pride Festival to support LGBT+ rights. During this period, it is likely that the number of contacts between men who have sex with men (MSM) increased, further affecting the transmission rate. However, after the end of Pride day, the number of contacts within high- risk groups gradually decreased. This reduction in exposure may be attributed to the media coverage of mpox outbreaks and public information campaigns on disease control measures, which influenced human behaviour [30]. The contact probability matrix c_{ij}^{hh} within the high-risk group can be obtain by the following Equation (7)

$$c_{ij}^{hh} = \tau_i \chi_{ij} + (1 - \tau_i) g_j, \quad g_j = \frac{(1-\tau_j)\alpha_j^{hh} N_j^h}{\sum_k (1-\tau_k)\alpha_k^{hh} N_k^h}, \tag{7}$$

where $\tau_i \in [0\ 1]$ are parameters that characterize preference for contact within the same age group and χ_{ij} is the Kronecker delta function (χ_{ij}=1 when $i = j$ and 0 otherwise) [29]. According to [31], the homosexual males are more likely to seek partners of similar age. Hence, we let $\tau_i = 0.5,\ (0 \leq i \leq 5)$. The contact matrix among age groups is shown in Figure S6. The contact matrices for low-risk population to low-risk population $C^{ll} = \alpha^{ll} c_0^{ll}$ and low-risk to high-risk population $C_i^{hl} = \alpha_i^{hl} c_i^{hl}$ are not sensitive to the epidemic scale of mpox. Hence, they are assumed to be constants and are captured in [32, 33]. The average number of contacts between a high-risk individual and low-risk individuals $\alpha^{lh} = \alpha^{lh}(t)$ is estimated from the Model (5) by MCMC. The contact probability matrix ($c_i^{lh} = [0.23\ 0.29\ 0.19\ 0.14\ 0.13]$) of high-risk individuals contacting with low-risk individuals is influenced by the age distribution within the high-risk population.

3.2.3. *Vaccination Parameters*

Combined with the actual vaccination data (Table S2), we can obtain the effective vaccination rates as shown in Table S4. These are determined by Equation

$$\bar{\sigma}_i^h(t) = \frac{Does_i^h(t)}{S_i^h(t)},\ \tilde{\sigma}_i^h(t) = \frac{Does_i^h(t)}{\tilde{V}_i^h(t)},$$

$$\bar{\sigma}^l(t) = \frac{Does^l(t)}{S^l(t)},\ \tilde{\sigma}_i^h(t) = \frac{Does^l(t)}{\tilde{V}^l(t)},$$

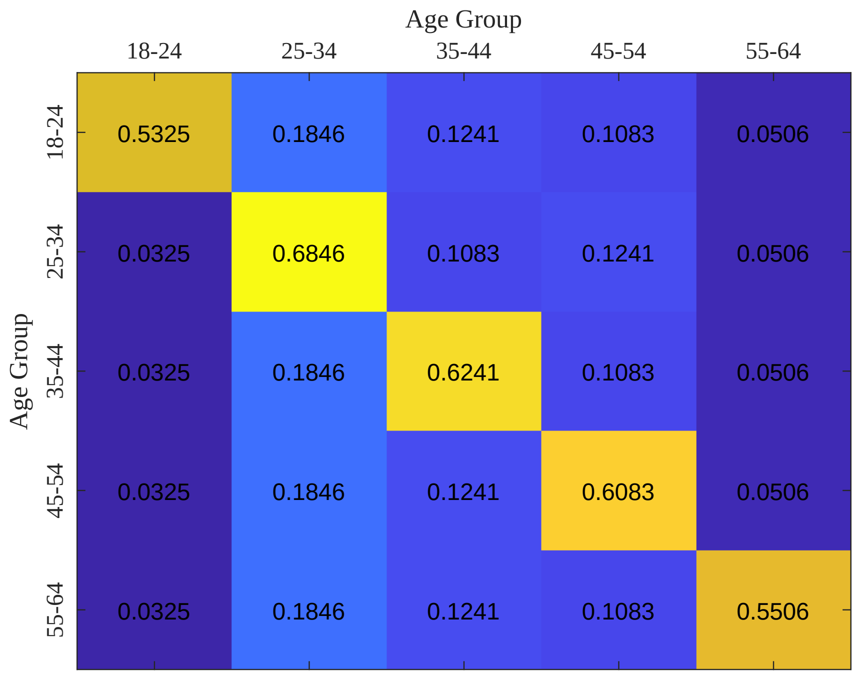

Figure S6: The contact matrix of each age group comes from Equation (7) [29].

where $Dose_i^h$ and $Dose^l$ are the vaccination doses for the i-th age group in the high-risk population and the all age groups in the low-risk population.

3.3. *Fitting Result*

The MCMC method is used to fit Model (5) for 800000 iterations with a burn-in of 640001 iterations. The traces plots of unknown parameters and initial values for System (5) are shown in Figure S8. The weekly new cases in New York City are used to estimate unknown parameters and initial values. The time step is week in the simulations. For the loss function, we take the mean square type. The numerical simulation results for different population and age groups are shown in Figure S7.

4. Effective Reproduction Number

The effective reproduction number is an important indicator in infectious disease models, measuring the direction of the epidemic, that is, the outbreak or disappearance.

4.1. *Theoretical Derivation*

The basic reproduction number, denoted by R_0, is the average number of an infected individuals can infect without intervention and with all susceptible individuals [34]. We use the next-generation matrix approach to derive R_0 [35]. In high-risk populations, the rate of new infection in age group i, denoted by

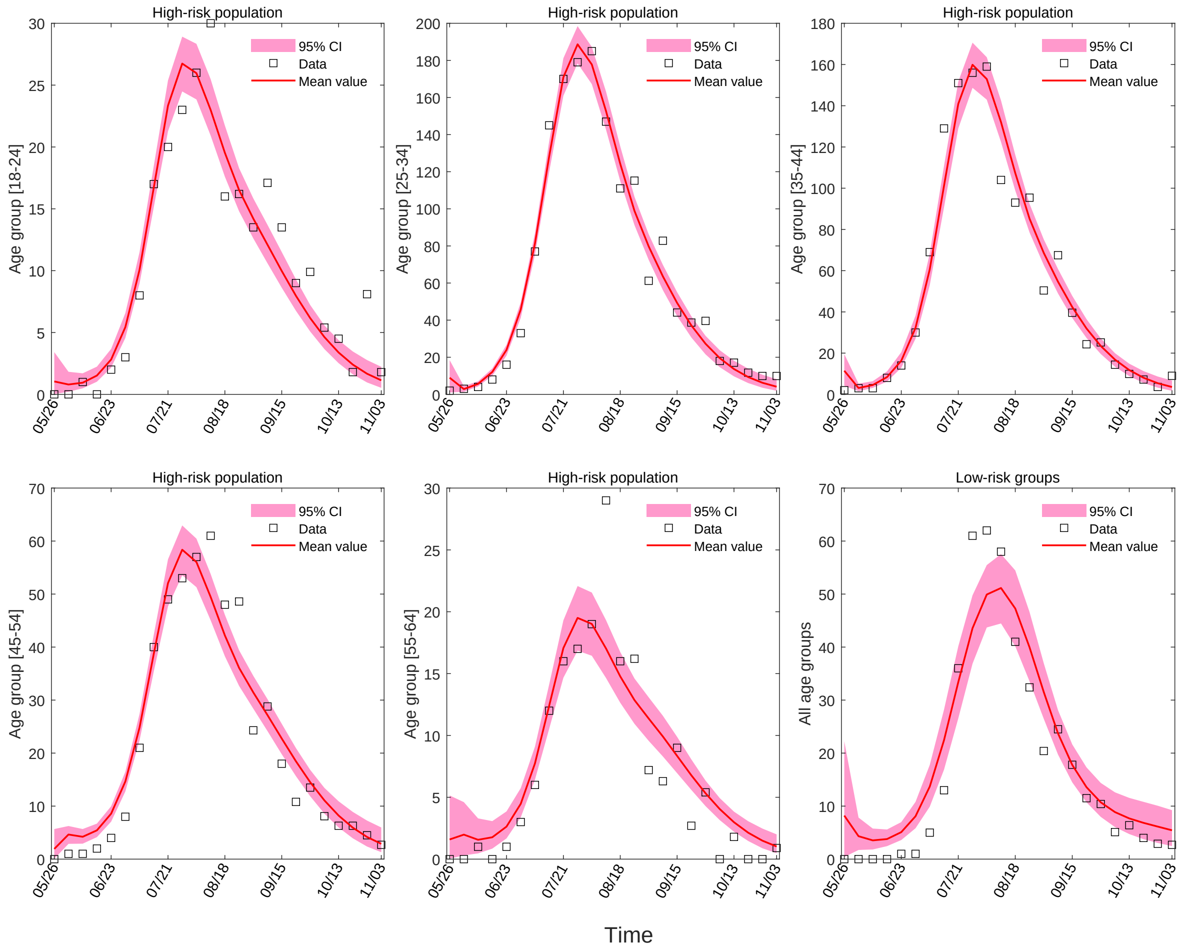

Figure S7: Fitted results of the number of new weekly mpox cases from May 19 to November 3. The solid red line shows the fitted curve of the Model (5). The 95% confidence interval is indicated in light red. The white squares represent the actual number of new weekly cases.

$\mathcal{F}_i^h$ and the rate of internal evolution in age group i, denoted by $\mathcal{V}_i^h$ are as follows

$$\mathcal{F}_i^h = \begin{bmatrix} \alpha_i^{hh} \beta^h \sum_{j=1}^{n} c_{ij}^h \left(\frac{\epsilon_j^h P_j^h + I_j^h}{N_j^h}\right)(S_i^h + \tilde{\delta}_i^h \tilde{V}_i^h + \bar{\delta}_i^h \bar{V}_i^h) + \alpha_i^{hl} \beta^l c_i^{hl} \left(\frac{\epsilon^l P^l + I^l}{N^l}\right)(S_i^h + \tilde{\delta}_i^h \tilde{V}_i^h + \bar{\delta}_i^h \bar{V}_i^h) \\ 0 \\ 0 \end{bmatrix}, \tag{8}$$

$$\mathcal{V}_i^h = \begin{bmatrix} \eta_i^h E_i^h \\ -\eta_i^h E_i^h + \mu_i^h P_i^h \\ -\mu_i^h P_i^h + \gamma_i^h I_i^h \end{bmatrix}, \tag{9}$$

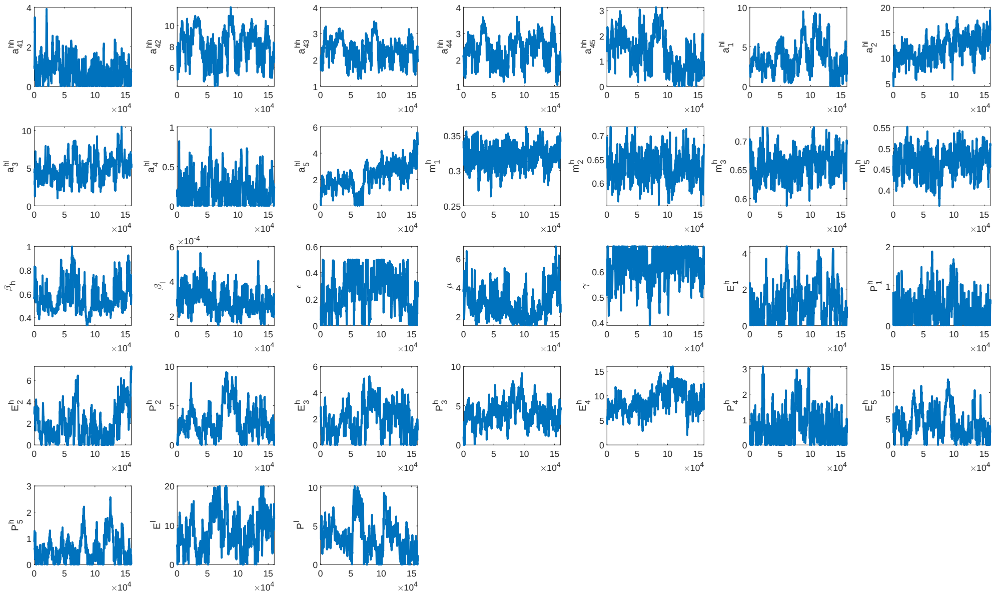

Figure S8: Trace plots of unknown parameters and initial values for System (5), estimated by Markov chain Monte Carlo (MCMC) methods.

Similarly, in the low-risk population, we get

$$\mathcal{F}^l = \begin{bmatrix} \alpha^{lh}\beta^l \sum_{j=1}^{n} c_j^{lh} \left(\frac{\epsilon_j^h P_j^h + I_j^h}{N_j^h}\right)(S^l + \tilde{\delta}^l \tilde{V}^l + \bar{\delta}^l \bar{V}^l) + \alpha^{ll}\beta^l c_0^l \left(\frac{\epsilon^l P^l + I^l}{N^l}\right)(S^l + \tilde{\delta}^l \tilde{V}^l + \bar{\delta}^l \bar{V}^l) \\ 0 \\ 0 \end{bmatrix}, \tag{10}$$

$$\mathcal{V}_i^h = \begin{bmatrix} \eta^l E^l \\ -\eta^l E^l + \mu^l P^l \\ -\mu^l P^l + \gamma^l I^l \end{bmatrix}. \tag{11}$$

The Jacobin matrices of $\mathcal{F}_i^h$ and $\mathcal{F}^l$ at disease free equilibrium:

$$(S_1^h(0), \tilde{V}_1^h(0), \bar{V}_1^h(0), 0, 0, 0, 0, \cdots, S_n^h(0), \tilde{V}_n^h(0), \bar{V}_n^h(0), 0, 0, 0, 0, \bar{S}^l(0), \tilde{V}^l(0), \bar{V}^l(0), 0, 0, 0, 0)$$

are $F_i^h =$

$$\begin{bmatrix} 0 & \alpha_i^{hh}\frac{\beta^h c_{i1}^h \epsilon_1^h}{N_1^h} Z_i^h(0) & \alpha_i^{hh}\frac{\beta^h c_{i1}^h}{N_1^h} Z_i^h(0) & \cdots & 0 & \alpha_i^{hh}\frac{\beta^h c_{in}^h \epsilon_n^h}{N_n^h} Z_i^h(0) & \alpha_i^{hh}\frac{\beta^h c_{in}^h}{N_n^h} Z_i^h(0) & 0 & \alpha_i^{hl}\frac{\beta^l c_i^{hl} \epsilon^l}{N^l} Z_i^h(0) & \alpha_i^{hl}\frac{\beta^l c_i^{hl}}{N^l} Z_i^h(0) \\ 0 & 0 & 0 & \cdots & 0 & 0 & 0 & 0 & 0 & 0 \\ 0 & 0 & 0 & \cdots & 0 & 0 & 0 & 0 & 0 & 0 \end{bmatrix},$$

and $F^l =$

$$\begin{bmatrix} 0 & \alpha^{lh}\frac{\beta^l c_1^{lh} \epsilon_1^h}{N_1^h} Z^l(0) & \alpha^{lh}\frac{\beta^l c_1^{lh}}{N_1^h} Z^l(0) & \cdots & 0 & \alpha^{lh}\frac{\beta^l c_n^{lh} \epsilon_n^h}{N_n^h} Z^l(0) & \alpha^{lh}\frac{\beta^l c_n^{lh}}{N_n^h} Z^l(0) & 0 & \alpha^{ll}\frac{\beta^l c_0^l \epsilon^l}{N^l} Z^l(0) & \alpha^{ll}\frac{\beta^l c_0^l}{N^l} Z^l(0) \\ 0 & 0 & 0 & \cdots & 0 & 0 & 0 & 0 & 0 & 0 \\ 0 & 0 & 0 & \cdots & 0 & 0 & 0 & 0 & 0 & 0 \end{bmatrix},$$

where

$$Z_i^h(0) := S_i^h(0) + \tilde{\delta}_i^h \tilde{V}_i^h(0) + \bar{\delta}_i^h \bar{V}_i^h(0), \quad Z^l(0) := S^l(0) + \tilde{\delta}^l \tilde{V}^l(0) + \bar{\delta}^l \bar{V}^l(0).$$

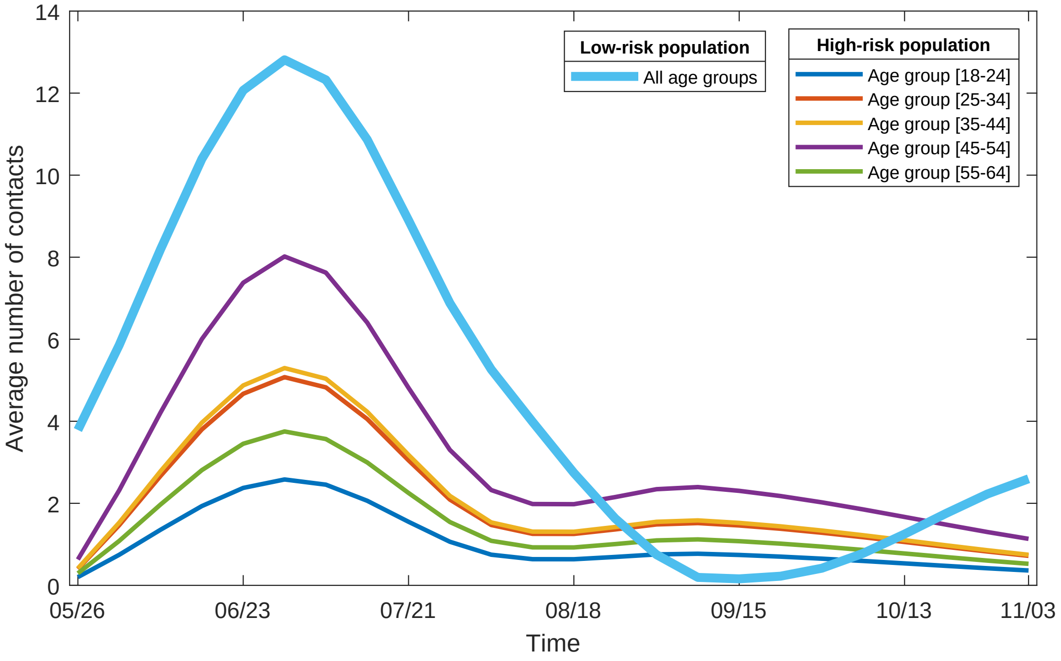

Figure S9: The average number of contacts in different groups. a_1^{hh}, a_2^{hh}, a_3^{hh}, a_4^{hh}, a_5^{hh} represents the average contacts number in high-risk groups aged 18-24 years old, 25-34 years old, 35-44 years old, 45-54 years old and 55-64 years old, respectively. a^{hl} is the average number of contacts of low-risk population to high-risk population.

$$F := \begin{bmatrix} F_1^h \\ \vdots \\ F_n^h \\ F^l \end{bmatrix}, := \begin{bmatrix} f_{11} & \cdots & f_{1n} & f_{1n+1} \\ \vdots & \vdots & \vdots & \vdots \\ f_{n1} & \cdots & f_{nn} & f_{nn+1} \\ f_{n+11} & \cdots & f_{n+1n} & f_{n+1n+1} \end{bmatrix}.$$

Hence, the Jacobian matrices representing the numbers of new infection for all groups are

$$f_{ij} = \begin{bmatrix} 0 & \alpha_i^{hh} \frac{\beta^h c_{ij}^h \epsilon_j^h}{N_j^h} Z_i^h(0) & \alpha_i^{hh} \frac{\beta^h c_{ij}^h}{N_j^h} Z_i^h(0) \\ 0 & 0 & 0 \\ 0 & 0 & 0 \end{bmatrix}, \text{where } i, j = 1, \cdots, n.$$

$$f_{in+1} = \begin{bmatrix} 0 & \alpha_i^{hl} \frac{\beta^l c_i^{hl} \epsilon^l}{N^l} Z^l(0) & \alpha_i^{hl} \frac{\beta^l c_i^{hl}}{N^l} Z^l(0) \\ 0 & 0 & 0 \\ 0 & 0 & 0 \end{bmatrix}, \text{where } i = 1, \cdots, n.$$

$$f_{n+1j} = \begin{bmatrix} 0 & \alpha^{lh} \frac{\beta^l c_j^{lh} \epsilon_j^h}{N_j^h} Z^l(0) & \alpha^{ll} \frac{\beta^l c_j^{lh}}{N_j^h} Z^l(0) \\ 0 & 0 & 0 \\ 0 & 0 & 0 \end{bmatrix}, \text{where } j = 1, \cdots, n.$$

$$f_{n+1n+1} = \begin{bmatrix} 0 & \alpha^{ll} \frac{\beta^l c_0^l \epsilon^l}{N^l} Z^l(0) & \alpha^{ll} \frac{\beta^l c_0^l}{N^l} Z^l(0) \\ 0 & 0 & 0 \\ 0 & 0 & 0 \end{bmatrix}.$$

For $\mathcal{V}^h$ and $\mathcal{V}^l$, the Jacobin matrices at

$$(\bar{S}_1^h, \bar{V}_1^h, 0, 0, 0, 0, \cdots, \bar{S}_i^h, \bar{V}_i^h, 0, 0, 0, 0, \cdots, \bar{S}_n^h, \bar{V}_n^h, 0, 0, 0, 0, \bar{S}^l, \bar{V}^l, 0, 0, 0, 0)$$

are

$$V_i^h = \begin{bmatrix} 0 & 0 & 0 & \cdots & \eta_i^h & 0 & 0 & \cdots & 0 & 0 & 0 & 0 & 0 & 0 \\ 0 & 0 & 0 & \cdots & -\eta_i^h & \mu_i^h & 0 & \cdots & 0 & 0 & 0 & 0 & 0 & 0 \\ 0 & 0 & 0 & \cdots & 0 & -\mu_i^h & \gamma_i^h & \cdots & 0 & 0 & 0 & 0 & 0 & 0 \end{bmatrix},$$

$$V^l = \begin{bmatrix} 0 & 0 & 0 & \cdots & 0 & 0 & 0 & \cdots & 0 & 0 & 0 & \eta^l & 0 & 0 \\ 0 & 0 & 0 & \cdots & 0 & 0 & 0 & \cdots & 0 & 0 & 0 & -\eta^l & \mu^l & 0 \\ 0 & 0 & 0 & \cdots & 0 & 0 & 0 & \cdots & 0 & 0 & 0 & 0 & -\mu^l & \gamma^l \end{bmatrix}.$$

We let

$$V := \begin{bmatrix} V_1^h \\ \vdots \\ V_n^h \\ V^l \end{bmatrix} := \begin{bmatrix} v_{11} & \cdots & v_{1n} & v_{1n+1} \\ \vdots & \vdots & \vdots & \vdots \\ v_{n1} & \cdots & v_{nn} & v_{nn+1} \\ v_{n+11} & \cdots & v_{n+1n} & v_{n+1n+1} \end{bmatrix}.$$

For $i = j$,

$$v_{ij} = \begin{bmatrix} \eta_i^h & 0 & 0 \\ -\eta_i^h & \mu_i^h & 0 \\ 0 & -\mu_i^h & \gamma_i^h \end{bmatrix}.$$

For $i \neq j$, $v_{ij} = 0$. Additionally,

$$v_{n+1n+1} = \begin{bmatrix} \eta^l & 0 & 0 \\ -\eta^l & \mu^l & 0 \\ 0 & -\mu^l & \gamma^l \end{bmatrix}.$$

Therefore,

$$V = \begin{bmatrix} v_{11} & \cdots & 0 & 0 \\ \vdots & \vdots & \vdots & \vdots \\ 0 & \cdots & v_{nn} & 0 \\ 0 & \cdots & 0 & v_{n+1n+1} \end{bmatrix}.$$

The next-generation matrix is

$$FV^{-1} = \begin{bmatrix} f_{11}v_{11}^{-1} & \cdots & f_{1n}v_{nn}^{-1} & f_{1n+1}v_{n+1n+1}^{-1} \\ \vdots & \vdots & \vdots & \vdots \\ f_{n1}v_{11}^{-1} & \cdots & f_{nn}v_{nn}^{-1} & f_{nn+1}v_{n+1n+1}^{-1} \\ f_{n+11}v_{11}^{-1} & \cdots & f_{n+1n}v_{n+1n}^{-1} & f_{n+1n+1}v_{n+1n+1}^{-1} \end{bmatrix}.$$

The basic reproduction number is $R_0 = \rho(FV^{-1})$, where $\rho(\cdot)$ means the dominant eigenvalue. The effective reproduction number predicts the average number of secondary confirmed cases produced by an infected individual diagnosed at time t during the infection period. The effective reproduction number R_e of the Model (5) is $\rho(F_t V^{-1})$, where $F_t =$

$$\begin{bmatrix} 0 & \alpha_1^{hh}\frac{\beta^h c_{11}^h \epsilon_1^h}{N_1^h} Z_1^h(t) & \alpha_1^{hh}\frac{\beta^h c_{11}^h}{N_1^h} Z_1^h(t) & \cdots & 0 & \alpha_1^{hh}\frac{\beta^h c_{1n}^h \epsilon_n^h}{N_n^h} Z_1^h(t) & \alpha_1^{hh}\frac{\beta^h c_{1n}^h}{N_n^h} Z_1^h(t) & 0 & \alpha_1^{hl}\frac{\beta^l c_1^{hl} \epsilon^l}{N^l} Z_1^h(t) & \alpha_1^{hl}\frac{\beta^l c_1^{hl}}{N^l} Z_1^h(t) \\ 0 & 0 & 0 & \cdots & 0 & 0 & 0 & 0 & 0 & 0 \\ 0 & 0 & 0 & \cdots & 0 & 0 & 0 & 0 & 0 & 0 \\ \vdots & \vdots & \vdots & \vdots & \vdots & \vdots & \vdots & \vdots & \vdots & \vdots \\ 0 & \alpha_i^{hh}\frac{\beta^h c_{n1}^h \epsilon_1^h}{N_1^h} Z_n^h(t) & \alpha_n^{hh}\frac{\beta^h c_{n1}^h}{N_1^h} Z_n^h(t) & \cdots & 0 & \alpha_n^{hh}\frac{\beta^h c_{nn}^h \epsilon_n^h}{N_n^h} Z_n^h(t) & \alpha_n^{hh}\frac{\beta^h c_{nn}^h}{N_n^h} Z_n^h(t) & 0 & \alpha_n^{hl}\frac{\beta^l c_n^{hl} \epsilon^l}{N^l} Z_n^h(t) & \alpha_n^{hl}\frac{\beta^l c_n^{hl}}{N^l} Z_n^h(t) \\ 0 & 0 & 0 & \cdots & 0 & 0 & 0 & 0 & 0 & 0 \\ 0 & 0 & 0 & \cdots & 0 & 0 & 0 & 0 & 0 & 0 \\ 0 & \alpha^{lh}\frac{\beta^l c_1^{lh} \epsilon_1^h}{N_1^h} Z^l(t) & \alpha^{lh}\frac{\beta^l c_1^{lh}}{N_1^h} Z^l(t) & \cdots & 0 & \alpha^{lh}\frac{\beta^l c_n^{lh} \epsilon_n^h}{N_n^h} Z^l(t) & \alpha^{lh}\frac{\beta^l c_n^{lh}}{N_n^h} Z^l(t) & 0 & \alpha^{ll}\frac{\beta^l c_0^l \epsilon^l}{N^l} Z^l(t) & \alpha^{ll}\frac{\beta^l c_0^l}{N^l} Z^l(t) \\ 0 & 0 & 0 & \cdots & 0 & 0 & 0 & 0 & 0 & 0 \\ 0 & 0 & 0 & \cdots & 0 & 0 & 0 & 0 & 0 & 0 \end{bmatrix}.$$

4.2. Effective Reproduction Number For New York City

From the estimated parameters, we calculate the effective reproduction number $Re(t)$, see Figure S10. Under the effect of control strategies (vaccine and behavioral control), mpox will break out when $Re > 1$ and become extinct when $Re < 1$.

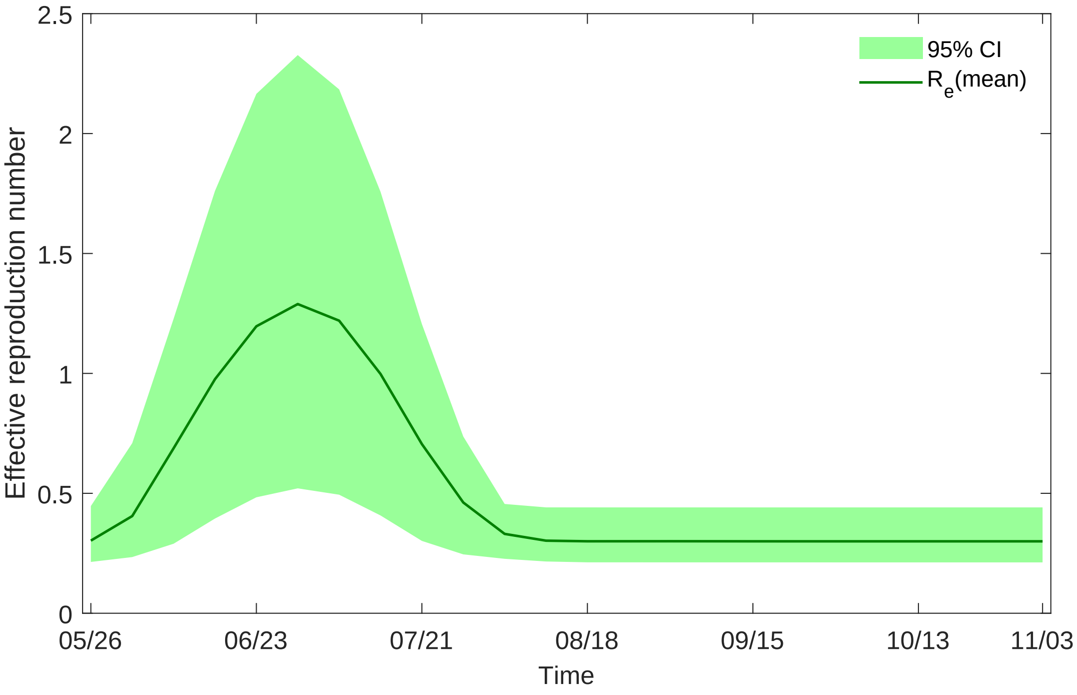

Figure S10: The numerical result of the effective reproduction number.

5. Sensitivity Analysis

We use the LHS (Latin hypercube sampling) and the PRCCs (partial rank correlation coefficients) to study the global uncertainty and sensitivity of the effective vaccination rate. Our goal is to find the key vaccination parameters that impact effective reproduction number R_e and cumulative number of new cases. We selected the mean of effective vaccination rate according to Table S4. The variance of the effective vaccination rate is set at 0.1 times of the mean. The sensitivity results for different effective vaccination rates are shown in Figure S11.

Acknowledgements

LX is funded by the National Natural Science Foundation of China 12171116 and Fundamental Research Funds for the Central Universities 3072020CFT2402 and 3072022TS2404.

References

[1] Anderson, R. M., & May, R. M. (1991). Infectious diseases of humans: dynamics and control. Oxford university press.

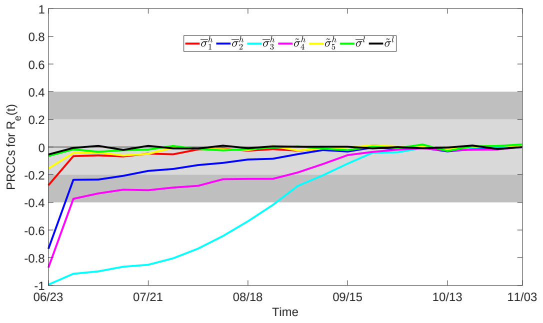

(a)

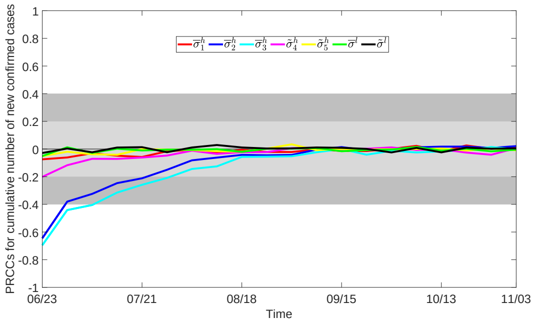

(b)

Figure S11: Results of sensitivity analysis on effective reproduction number and the cumulative number of new cases about vaccination rate. The results showed that vaccination of men who had sex with men was more sensitive to reducing new cases. In addition, the sensitivity to vaccination in terms of age group is $[35-44] > ([45-54]$ or $[25-34]) > [18-24] > [64+]$. In terms of vaccination timing, vaccination before 06/23 is shown to be most effective, that is, before the large-scale outbreak of the epidemic.

[2] Wormser, G. P., & Pourbohloul, B. (2008). Modeling Infectious Diseases in Humans and Animals, NJ: Princeton University Press, 2008.

[3] Bhunu, C. P., & Mushayabasa, S. (2011). Modelling the transmission dynamics of pox-like infections.

[4] Usman, S., & Adamu, I. I. (2017). Modeling the transmission dynamics of the monkeypox virus infection with treatment and vaccination interventions. Journal of Applied Mathematics and Physics, 5(12), 2335.

[5] Somma, S. A., Akinwande, N. I., & Chado, U. D. (2019). A mathematical model of monkey pox virus transmission dynamics. Ife Journal of Science, 21(1), 195-204.

[6] TeWinkel, R. E. (2019). Stability analysis for the equilibria of a monkeypox model. The University of Wisconsin-Milwaukee.

[7] Peter, O. J., Kumar, S., Kumari, N., Oguntolu, F. A., Oshinubi, K., & Musa, R. (2022). Transmission dynamics of Monkeypox virus: a mathematical modelling approach. Modeling Earth Systems and Environment, 1-12.

[8] Grant, R., Nguyen, L. B. L., & Breban, R. (2020). Modelling human-to-human transmission of monkeypox. Bulletin of the World Health Organization, 98(9), 638.

[9] Betti, M. I., Farrell, L., & Heffernan, J. (2022). A pair formation model with recovery: Application to Monkeypox. medRv, 2022-08.

[10] Bubar, K. M., Reinholt, K., Kissler, S. M., Lipsitch, M., Cobey, S., Grad, Y. H., & Larremore, D. B. (2021). Model-informed COVID-19 vaccine prioritization strategies by age and serostatus. Science, 371(6352), 916-921.

[11] Xue, L., Jing, S., & Wang, H. (2022). Evaluating strategies for tuberculosis to achieve the goals of WHO in China: a seasonal age-structured model study. Bulletin of Mathematical Biology, 84(6), 61.

[12] Bartlett, J. G. (2004). The detection of monkeypox in humans in the western hemisphere. Infectious Diseases in Clinical Practice, 12(4), 275-276.

[13] Thornhill, J. P., Barkati, S., Walmsley, S., Rockstroh, J., Antinori, A., Harrison, L. B., & Orkin, C. M. (2022). Monkeypox virus infection in humans across 16 countries-April-June 2022. New England Journal of Medicine, 387(8), 679-691.

[14] World Health Organization, 2022 mpox Outbreak: Global Trends. Available from: https://worldhealthorg.shinyapps.io/mpx_global/. Accessed Nov 17, 2022.

[15] World Health Organization, mpox. Available from: https://www.who.int/news-room/questions-and-answers/item/monkeypox. Accessed Nov 3, 2022.

[16] Amanna, I. J., Carlson, N. E., & Slifka, M. K. (2007). Duration of humoral immunity to common viral and vaccine antigens. New England Journal of Medicine, 357(19), 1903-1915.

[17] De Baetselier, I., Van Dijck, C., Kenyon, C., Coppens, J., Michiels, J., de Block, T., & Van Esbroeck, M. (2022). Retrospective detection of asymptomatic monkeypox virus infections among male sexual health clinic attendees in Belgium. Nature Medicine, 28(11), 2288-2292.

[18] World Health Network, Monkeypox meter, Available from: https://www.monkeypoxmeter.com/. Accessed Aug 23, 2023.

[19] HYC health, mpox. Available from: https://www.nyc.gov/site/doh/health/health-topics/monkeypox.page. Accessed Nov 3, 2022.

[20] World Population Review, New York City Population. Available from: https://worldpopulationsreview.com/us-cities/new-york-city-ny-population. Accessed Nov 3, 2022.

[21] UCLA school of law williams institute, LGBT Data & Demographics Switch Topic. Available from: https://williamsinstitute.law.ucla.edu/visualization/lgbt-stats/?topic=LGBT&area=36&characteristic\=male#density. Accessed Nov 3, 2022.

[22] Centers for Disease Control and Prevention, History of Smallpox. Available from: https://www.cdc.gov/smallpox/history/history.html. Accessed Nov 3, 2022.

[23] Hammarlund, E., Lewis, M. W., Hansen, S. G., Strelow, L. I., Nelson, J. A., Sexton, G. J., & Slifka, M. K. (2003). Duration of antiviral immunity after smallpox vaccination. Nature Medicine, 9(9), 1131-1137.

[24] Miura, F., van Ewijk, C. E., Backer, J. A., Franz, E., de Coul, E. O., & Wallinga, J. (2022). Estimated incubation period for monkeypox cases confirmed in the Netherlands, May 2022. Eurosurveillance, 27(24), 2200448.

[25] Guzzetta, G., Mammone, A., Ferraro, F., Caraglia, A., Rapiti, A., Marziano, V., & Merler, S. (2022). Early estimates of monkeypox incubation period, generation time, and reproduction number, Italy, MayCJune 2022. Emerging Infectious Diseases, 28(10), 2078.

[26] Gomez-Garberi, M., Sarrio-Sanz, P., Martinez-Cayuelas, L., Delgado-Sanchez, E., Bernabeu-Cabezas, S., Peris-Garcia, J., & Ortiz-Gorraiz, M. A. (2022). Genitourinary lesions due to monkeypox. European Urology, 82(6), 625-630.

[27] Amanna, I. J., Carlson, N. E., & Slifka, M. K. (2007). Duration of humoral immunity to common viral and vaccine antigens. New England Journal of Medicine, 357(19), 1903-1915.

[28] Haider, N., Guitian, J., Simons, D., Asogun, D., Ansumana, R., Honeyborne, I., & Zumla, A. (2022). Increased outbreaks of monkeypox highlight gaps in actual disease burden in Sub-Saharan Africa and in animal reservoirs. International Journal of Infectious Diseases, 122, 107-111.

[29] Feng, Z., Feng, Y., & Glasser, J. W. (2020). Influence of demographically-realistic mortality schedules on vaccination strategies in age-structured models. Theoretical Population Biology, 132, 24-32.

[30] Centers for Disease Control and Prevention, Impact of mpox Outbreak on Select Behaviors. Available from: https://www.cdc.gov/poxvirus/monkeypox/response/2022/amis-select-behaviors.html. Accessed Nov 3, 2022.

[31] Harry, J. (1982). Decision making and age differences among gay male couples. Journal of Homosexuality, 8(2), 9-21.

[32] Prem, K., Cook, A. R., & Jit, M. (2017). Projecting social contact matrices in 152 countries using contact surveys and demographic data. PLoS Computational Biology, 13(9), e1005697.

[33] Jacquez, J. A., Simon, C. P., Koopman, J., Sattenspiel, L., & Perry, T. (1988). Modeling and analyzing HIV transmission: the effect of contact patterns. Mathematical Biosciences, 92(2), 119-199.

[34] Diekmann, O., Heesterbeek, J. A. P., & Metz, J. A. (1990). On the definition and the computation of the basic reproduction ratio R_0 in models for infectious diseases in heterogeneous population. Journal of Mathematical Biology, 28, 365-382.

[35] Cui, J., Wu, Y., & Guo, S. (2022). Effect of non-homogeneous mixing and asymptomatic individuals on final epidemic size and basic reproduction number in a meta-population model. Bulletin of Mathematical Biology, 84(3), 38.

Table S3: Initial Value for parameter fitting

**Symbol**	**Interpretation**	**Value**	**Source**
N	Total population of New York City	8930002	[20]
N^h	Number of high-risk population in New York City	127030	[21]
N_1^h	Number of high-risk population aged 18-24 years in New York City	16001	[21]
$S_1^h(0)$	Initial number of susceptible individuals aged 18-24 years in high-risk population	16000	Calculated
$\tilde{V}_1^h(0)$	Initial number of vaccinated individuals aged 18-24 years in high-risk population	0	[22, 23]
$\bar{V}_1^h(0)$	Initial number of vaccinated individuals aged 18-24 years in high-risk population	0	[22, 23]
$E_1^h(0)$	Initial number of exposed individuals aged 18-24 years in high-risk population	0	MCMC
$P_1^h(0)$	Initial number of prodromal individuals aged 18-24 years in high-risk population	1	MCMC
$I_1^h(0)$	Initial number of infected individuals aged 18-24 years in high-risk population	0	Data
$R_1^h(0)$	Initial number of recovered individuals aged 18-24 years in high-risk population	0	Estimated
N_2^h	Number of high-risk population aged 25-34 years in New York City	45962	[21]
$S_2^h(0)$	Initial number of susceptible individuals aged 25-34 years in high-risk population	45953	Calculated
$\tilde{V}_2^h(0)$	Initial number of vaccinated individuals aged 25-34 years in high-risk population	0	[22, 23]
$\bar{V}_2^h(0)$	Initial number of vaccinated individuals aged 25-34 years in high-risk population	0	[22, 23]
$E_2^h(0)$	Initial number of exposed individuals aged 25-34 years in high-risk population	7	MCMC
$P_2^h(0)$	Initial number of prodromal individuals aged 25-34 years in high-risk population	0	MCMC
$I_2^h(0)$	Initial number of infected individuals aged 25-34 years in high-risk population	2	Data
$R_2^h(0)$	Initial number of recovered individuals aged 25-34 years in high-risk population	0	Estimated
N_3^h	Number of high-risk population aged 35-44 years in New York City	30054	[21]
$S_3^h(0)$	Initial number of susceptible individuals aged 35-44 years in high-risk population	30047	Calculated
$\tilde{V}_3^h(0)$	Initial number of vaccinated individuals aged 35-44 years in high-risk population	0	[22, 23]
$\bar{V}_3^h(0)$	Initial number of vaccinated individuals aged 35-44 years in high-risk population	0	[22, 23]
$E_3^h(0)$	Initial number of exposed individuals aged 35-44 years in high-risk population	1	MCMC
$P_3^h(0)$	Initial number of prodromal individuals aged 35-44 years in high-risk population	4	MCMC
$I_3^h(0)$	Initial number of infected individuals aged 35-44 years in high-risk population	2	Data
$R_3^h(0)$	Initial number of recovered individuals aged 35-44 years in high-risk population	0	Estimated
N_4^h	Number of high-risk population aged 45-54 years in New York City	18210	[21]
$S_4^h(0)$	Initial number of susceptible individuals aged 45-54 years in high-risk population	1820	Calculated
$\tilde{V}_4^h(0)$	Initial number of vaccinated individuals aged 45-54 years in high-risk population	16380	[22, 23]
$\bar{V}_4^h(0)$	Initial number of vaccinated individuals aged 45-54 years in high-risk population	0	[22, 23]
$E_4^h(0)$	Initial number of exposed individuals aged 45-54 years in high-risk population	10	MCMC
$P_4^h(0)$	Initial number of prodromal individuals aged 45-54 years in high-risk population	0	MCMC
$I_4^h(0)$	Initial number of infected individuals aged 45-54 years in high-risk population	0	Data
$R_4^h(0)$	Initial number of recovered individuals aged 45-54 years in high-risk population	0	Estimated
N_5^h	Number of high-risk population aged 55-64 years in New York City	16802	[21]
$S_5^h(0)$	Initial number of susceptible individuals aged 55-64 years in high-risk population	1680	Calculated
$\tilde{V}_5^h(0)$	Initial number of vaccinated individuals aged 55-64 years in high-risk population	15120	[22, 23]
$\bar{V}_5^h(0)$	Initial number of vaccinated individuals aged 55-64 years in high-risk population	0	[22, 23]
$E_5^h(0)$	Initial number of exposed individuals aged 55-64 years in high-risk population	2	MCMC
$P_5^h(0)$	Initial number of prodromal individuals aged 55-64 years in high-risk population	0	MCMC
$I_5^h(0)$	Initial number of infected individuals aged 55-64 years in high-risk population	0	Data
$R_5^h(0)$	Initial number of recovered individuals aged 55-64 years in high-risk population	0	Estimated
N^l	Number of low-risk population in New York City	8802976	[20]
$S^l(0)$	Initial number of susceptible individuals in low-risk population	6024124	Calculated
$V^l(0)$	Initial number of vaccinated individuals in low-risk population	2778840	[22, 23]
$V^l(0)$	Initial number of vaccinated individuals in low-risk population	0	[22, 23]
$E^l(0)$	Initial number of exposed individuals in low-risk population	12	MCMC
$P^l(0)$	Initial number of prodromal individuals in low-risk population	0	MCMC
$I^l(0)$	Initial number of infected individuals in low-risk population	0	Data
$R^l(0)$	Initial number of recovered individuals in low-risk population	0	Estimated

Table S4: Effective vaccination rates in Model (5) corresponding to Table S2

Time	2022/05/26	2022/06/02	2022/06/09	2022/06/16	2022/06/23	2022/06/30	2022/07 /07	2022/07 /14	2022/07 /21
HRP*									
$\bar{\sigma}_1^h$	0	0	0	0	0.0014466107872	0.0036248177843	0.0087345116618	0.017875	0.035943285714
$\bar{\sigma}_2^h$	0	0	0	0	0.0020463296286	0.0051504646115	0.012420092807	0.025417274171	0.051101578942
$\bar{\sigma}_3^h$	0	0	0	0	0.0019441456199	0.0049041729667	0.011835898298	0.024228708357	0.048718417337
σ_4^h, $\bar{\sigma}_5^h$	0	0	0	0	0	0	0	0	0
σ_1^h, σ_2^h, $\tilde{\sigma}_3^h$	0	0	0	0	0	0	0	0	0
$\tilde{\sigma}_4^h$	0	0	0	0	0.0016289046231	0.0040885208086	0.0098539390639	0.020164835165	0.040543485086
$\tilde{\sigma}_5^h$	0	0	0	0	0.00147943758	0.0037114165394	0.0089492418283	0.01832010582	0.036845049131
LRP*									
$\tilde{\sigma}_5^h$ $(10^{-}4)$	0	0	0	0	0.00975324843	0.02348652782	0.055736646706	0.1134474199	0.22754565301
σ_5^h $(10^{-}4)$	0	0	0	0	0.010413044184	0.025075363728	0.059507165122	0.12112200406	0.24293884807

Time	2022/07 /21	2022/08/04	2022/08/11	2022/08/18	2022/08/25	2022/09/01	2022/09/08	2022/09/15
HRP*								
$\bar{\sigma}_1^h$	0.062353071429	0.089978714286	0.11169457143	0.120375	0.11120533333	0.088936	0.060789	0.033986333333
$\bar{\sigma}_2^h$	0.088639908478	0.12790531941	0.15877086837	0.17110961199	0.15807449641	0.12641681718	0.086407155137	0.048316091079
$\bar{\sigma}_3^h$	0.08451068564	0.12194966933	0.15137952446	0.1631444071	0.15071374846	0.12052477785	0.082373348421	0.046055313342
σ_4^h, $\bar{\sigma}_5^h$	0	0	0	0	0	0	0	0
σ_1^h, σ_2^h, $\tilde{\sigma}_3^h$	0	0	0	0	0	0	0	0
$\tilde{\sigma}_4^h$	0.070328728414	0.10148508634	0.12597708006	0.13576923077	0.12542813187	0.10031076923	0.068567252747	0.038347692308
$\tilde{\sigma}_5^h$	0.063922902494	0.092247543462	0.11451284958	0.12341269841	0.11400740741	0.091167724868	0.062303174603	0.034823280423
LRP*								
$\tilde{\sigma}_5^h$ $(10^{-}4)$	0.39432797813	0.56879294982	0.70593912262	0.76076505108	0.70286979262	0.56225431085	0.38452001968	0.21526833297
σ_5^h $(10^{-}4)$	0.4210038008	0.60727112207	0.75369507171	0.81222990957	0.75041810515	0.60029015188	0.41053234552	0.22983098182

Time	2022/09/22	2022/09/29	2022/10/06	2022/10/13	2022/10/20	2022/10/27	2022/11/03
HRP*							
$\bar{\sigma}_1^h$	0.01575	0.0070700231481	0.0030796296296	0.002215625	0.0029148148148	0.0036140046296	0.00275
$\bar{\sigma}_2^h$	0.022414205819	0.010111226769	0.0044859038744	0.0033123517507	0.0043646850123	0.0054170182739	0.004243466
$\bar{\sigma}_3^h$	0.021366525776	0.009652470389	0.0043117634225	0.0032199554032	0.0042525968575	0.0052852383118	0.00419343
σ_4^h, $\bar{\sigma}_5^h$	0	0	0	0	0	0	0
σ_1^h, σ_2^h, $\tilde{\sigma}_3^h$	0	0	0	0	0	0	0
$\tilde{\sigma}_4^h$	0.017802197802	0.0080498321123	0.0035982905983	0.0026806318681	0.0035299145299	0.0043791971917	0.003461538
$\tilde{\sigma}_5^h$	0.016137566138	0.0072631907701	0.0032069370958	0.0023627645503	0.0031246325691	0.0038865005879	0.003042328
LRP*							
$\tilde{\sigma}_5^h$ $(10^{-}4)$	0.10010066462	0.045266715156	0.020036610399	0.01454240211	0.018916142054	0.023289881999	0.017795674
σ_5^h $(10^{-}4)$	0.10687235652	0.048328955051	0.021392063462	0.015526178462	0.020195796755	0.024865415049	0.01899953

HRP: High-risk population, LRP: Low-risk population.

Table S5: Parameters value. Here i represents the i-th age group, and $0 \leq i \leq 5$.

Parameter	Value	Source	Parameter	Value	Source
β^h	0.56611	MCMC	β^l	0.00027	MCMC
$\bar{\delta}^l$, $\bar{\delta}^h_i$	0.15	[15]	$\tilde{\delta}^h_1$, $\tilde{\delta}^h_2$, $\tilde{\delta}^h_3$	1	[27]
$\tilde{\delta}^h_4$,	0.41	[27]	$\tilde{\delta}^h_5$	0.394402309	[27]
$\tilde{\delta}^l$	0.69	[27]	η^h_i	7/9.1	[25]
η^l	7/13	[26]	γ^l, γ^h_i	0.63	MCMC
ϵ^l, ϵ^h_i	0.275	MCMC	μ^l, μ^h_i	2.79	MCMC
a^{hh}_4	See Figure. S9	MCMC	a^{lh}	See Figure. S9	MCMC
m_1	0.32	MCMC	m_2	0.63	MCMC
m_3	0.65	MCMC	m_5	0.46	MCMC
a^{ll}	609	[32]	a^{hl}_1	57.4	[32]
a^{hl}_2	89.6	[21, 32]	a^{hl}_3	80.29	[21, 32]
a^{hl}_4	77.91	[21, 32]	a^{hl}_5	60.55	[21, 32]
c^{hh}	See Equation. (S6)	MCMC	c^{hl}_i	1	Estimated
c^{lh}_1	0.23	[20]	c^{lh}_2	0.29	[20]
c^{lh}_3	0.19	[20]	c^{lh}_4	0.14	[20]
c^{lh}_5	0.13	[20]	c^l_0	1	Estimated
$\bar{\sigma}^h_i$	See Table. S4	[19]	$\tilde{\sigma}^h_i$	See Table. S4	[19]
$\bar{\sigma}^l$	See Table. S4	[19]	$\tilde{\sigma}^l$	See Table. S4	[19]
